# The Role of ZO-2 in Modulating JAM-A and γ-Actin Junctional Recruitment, Apical Membrane and Tight Junction Tension, and Cell Response to Substrate Stiffness and Topography

**DOI:** 10.3390/ijms25052453

**Published:** 2024-02-20

**Authors:** Diana Cristina Pinto-Dueñas, Christian Hernández-Guzmán, Patrick Matthew Marsch, Anand Sunil Wadurkar, Dolores Martín-Tapia, Lourdes Alarcón, Genaro Vázquez-Victorio, Juan Vicente Méndez-Méndez, José Jorge Chanona-Pérez, Shikha Nangia, Lorenza González-Mariscal

**Affiliations:** 1 Department of Physiology, Biophysics and Neuroscience, Center for Research and Advanced Studies (Cinvestav), Mexico City 07360, Mexico; cristi_3289@cinvestav.mx (D.C.P.-D.); christian485@hotmail.com (C.H.-G.); dolores@fisio.cinvestav.mx (D.M.-T.); lalarcon@fisio.cinvestav.mx (L.A.); 2 Department of Biomedical and Chemical Engineering, Syracuse University, Syracuse, NY 13244, USA; pmarsch@syr.edu (P.M.M.); aswadurk@syr.edu (A.S.W.); snangia@syr.edu (S.N.); 3 Physics Department, Science School, National Autonomous University of Mexico (UNAM), Mexico City 04510, Mexico; genvazquez@ciencias.unam.mx; 4 Nanoscience, Micro and Nanotechnology Center, IPN, Mexico City 07738, Mexico; jmendezm@ipn.mx; 5 Department of Biochemical Engineering, ENCB, IPN, Mexico City 07738, Mexico; jchanona@ipn.mx

**Keywords:** ZO-2, tight junction, JAM-A, γ-actin, p114RhoGEF, afadin, tension

## Abstract

This work analyzes the role of the tight junction (TJ) protein ZO-2 on mechanosensation. We found that the lack of ZO-2 reduced apical membrane rigidity measured with atomic force microscopy, inhibited the association of γ-actin and JAM-A to the cell border, and instead facilitated p114RhoGEF and afadin accumulation at the junction, leading to an enhanced mechanical tension at the TJ measured by FRET, with a ZO-1 tension probe, and increased tricellular TJ tension. Simultaneously, adherens junction tension measured with an E-cadherin probe was unaltered. The stability of JAM-A and ZO-2 binding was assessed by a collaborative in silico study. The absence of ZO-2 also impacted the cell response to the substrate, as monolayers plated in 20 kPa hydrogels developed holes not seen in parental cultures and displayed a retarded elongation and formation of cell aggregates. The absence of ZO-2 was sufficient to induce YAP and Snail nuclear accumulation in cells cultured over glass, but when ZO-2 KD cells were plated in nanostructured ridge arrays, they displayed an increased abundance of nuclear Snail and conspicuous internalization of claudin-4. These results indicate that the absence of ZO-2 also impairs the response of cells to substrate stiffness and exacerbates transformation triggered by substrate topography.

## 1. Introduction

Epithelial cell layers cover the body surface and the lumen of body cavities and ducts. Mechanical strength between epithelial cells relies upon cell–cell adhesion provided by tight junctions (TJs), adherens junctions (AJ), and desmosomes. TJs are present at the uppermost part of the lateral membrane of epithelial cells [1] and have as canonical functions the regulation of the passage of ions and molecules through the paracellular pathway and the maintenance of cell polarity by blocking the free diffusion of lipids and proteins within the plasma membrane, from the apical to the basolateral surface and vice versa. TJs are associated with the perijunctional actomyosin cytoskeleton, allowing them to sense and respond to the mechanical forces affecting the epithelia (for review, see [2]). Here, we analyze the participation of the TJ adaptor protein Zonula occludens-2 (ZO-2) on mechanosensation, exploring if the lack of ZO-2 modulates the tension of the cell and its response to substrate stiffness and nanoscale topography.

ZO-2 is a member of the MAGUK protein family, containing three PDZ domains and SH3 and guanylate kinase (GuK) modules, followed by an actin-binding and proline-rich domain (for review, see [3]). ZO-2 directly interacts with actin [4,5], the integral TJ proteins claudins, occludin, and JAM-A, and the adaptor proteins afadin, cingulin, and ZO-1 (for review, see [3]). ZO-2 modulates the actomyosin cytoskeleton through the small Rho proteins RhoA, Rac, and CDC42. Thus, ZO-2 knock-down (KD) MDCK cells display increased activation of CDC42 and higher percentages of disoriented mitotic spindles, and when grown in three-dimensional cultures formed in the extracellular matrix Matrigel, they exhibit multiple lumens per cyst [6]. ZO-2 silencing also increases the activation of Rac and cofilin, inducing a profusion of lamellae and random cell migration, and of RhoA and Rho kinase 2 (ROCK-II), triggering the proliferation of stress fibers in MDCK cells [6]. Moreover, the complex formed by the interaction of JAM-A with afadin mediated by ZO-2 stimulates the activity of Rap2c, a GTPase that inhibits RhoA [7]. Likewise, the decreased expression of ZO-2 triggered in scirrhous cancer cells by treatment with TGFβ1 correlates with augmented RhoA activity and myosin phosphorylation [8].

MDCK cells that lack ZO-1 and ZO-2 display a prominent and highly organized ring of actin at the zonula adherens (ZA) with an altered punctuated distribution of nonmuscle myosin II (NMII). The elevated contractility of the actomyosin ring observed in these cells, which triggers an expansion of the apical domain [9], involves ROCK recruitment to the cell border mediated by Shroom3 [10]. 

A weak increase in apical tension measured by atomic force microscopy (AFM) has been observed in ZO-1 KD cells [11], and with non-contact acoustic frequency-modulation AFM, this increase has been found in the absence of both ZO-1 and ZO-2, while the inhibition of NMII activity with blebbistatin diminishes apical surface tension [12]. ZO-1 and ZO-2 are required to achieve normal apical tension, since the re-expression of only ZO-1 results in a partial decrease of the elevated tension present in the double KD cells [12]. In contrast, the AJ destruction by reducing the disulfide bridges of E-cadherin by treatment with 1,4-dithiothreitol (DTT) lowered the apical membrane tension [11]. 

The pattern of the cell border is also affected when ZO-1 [13,14] or both ZO-1 and ZO-2 [9] are silenced or knocked out, as a less wavy and more linear border appears, giving the cells a clear polygonal form. In contrast, cells overexpressing ZO-1 have an increased zigzag appearance of cell–cell junctions [14]. The increased linearity of the cell borders is also attained in control cells and ZO-1 KO cells when NMII is inhibited with blebbistatin, but is not observed when only ZO-2 is silenced or knocked out (KO) [9,14]. These observations suggest that although both ZO-1 and ZO-2 are associated with actin, their impact as TJ mechanosensors is different, prompting us to study the role played by ZO-2 in regulating the tension of the apical and lateral membranes. For this purpose, we have explored the apical tension in parental and ZO-2 KD MDCK cells with AFM, and their TJ and lateral membrane tension with FRET (fluorescence/Foerster resonance energy transfer), using a ZO-1 tension sensor and an E-cadherin tension sensor to measure this parameter at both the uppermost portion of the lateral membrane where TJs are located and bellow this region in the lateral membrane where the AJs are present. 

A previous study with FRET using the ZO-1 tension sensor showed that tension at the TJ augments with extracellular matrix stiffness [15], and numerous observations have revealed that cells grown on stiff matrices augment the number of focal adhesions and display an increase in cell spreading, proliferation, and migration [16,17]. Therefore, we have also investigated if the lack of ZO-2 affects the aggregation and spreading of MDCK cells plated on soft and stiff extracellular matrixes. In addition, we have explored if the lack of ZO-2 affects the expression of junctional proteins and nuclear factors in epithelial cells plated on matrixes with a nanoscale topography known to promote epithelial to mesenchymal transition (EMT)-like process [18]. 

Our results indicate that the lack of ZO-2 in epithelial cells decreases the apical membrane tension, which correlates with a reduced stability of microtubules and a diminished concentration of γ-actin at the TJ belt. In our in silico analysis, we characterized the molecular-level interactions of the ZO-2 PDZ-2 domain and JAM-A tail and computed the binding energy that leads to stable protein–protein association. Accordingly, the absence of ZO-2 in MDCK cells inhibits JAM-A association to the cell borders, facilitating p114RhoGEF and afadin junctional accumulation, leading to an enhanced mechanical tension at the TJ level measured with a ZO-1 tension probe. This increase in TJ tension was also detected by the recruitment of vinculin at tricellular TJs (tTJs) that run along the lateral membrane where three or more cells meet. Simultaneously, the lack of ZO-2 did not affect the tension at the AJ, measured with an E-cadherin tension probe. The increase in TJ tension triggered by the absence of ZO-2 also impacted how cells responded to the rigidity of the substrate, as monolayers of ZO-2 KD cells plated in 20 kPa hydrogels could not maintain stable monolayers and instead developed holes not seen in parental cultures. In addition, the absence of ZO-2 retarded cell elongation and the formation of cell aggregates in soft and stiff substrates covered with collagen IV or fibronectin. The lack of ZO-2 accentuated the response of the cells to substrate topography, as ZO-2 KD cells increased their internalization of claudin-4 and the accumulation at the nucleus of Snail and YAP when plated in nanostructured ridge arrays. 

## 2. Results

### 2.1. ZO-2 Depletion Decreases Apical Membrane Tension Modulated by Apical Microtubule Stability

**Figure 1:**
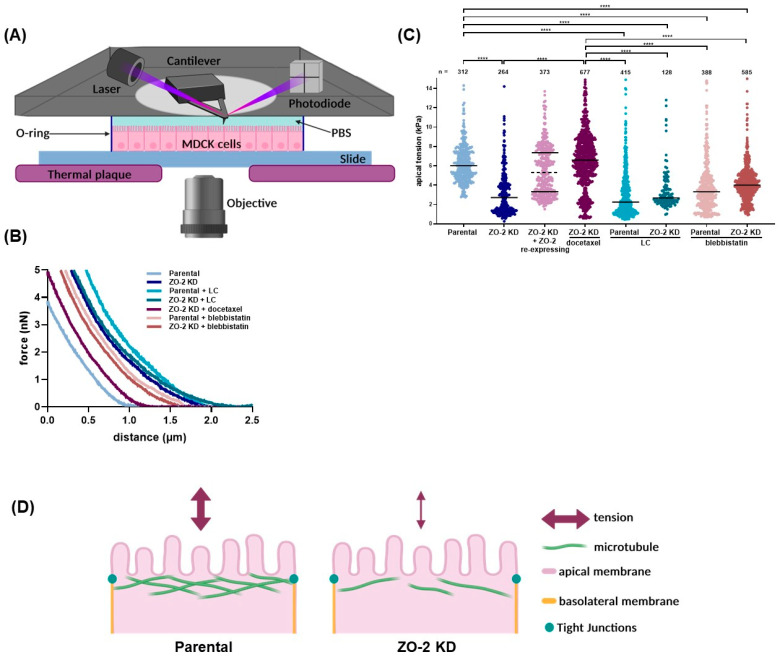
In MDCK cells, the lack of ZO-2 reduces the stiffness of the apical membrane. Apical membrane rigidity in kPa was determined using atomic force microscopy indentation in parental and ZO-2 KD monolayers incubated in low calcium (LC); in normal calcium conditions with or without blebbistatin 100 µM for 15 h prior to the experiment and through the indentation procedure; or, in the case of ZO-2 KD cells with 10 µM docetaxel, for 4 h prior to the experiment and through the indentation procedure. Apical membrane rigidity was also measured in monolayers of ZO-2 KD cells where ZO-2 was re-expressed in some cells scattered throughout the monolayer due to the lack of zeocin treatment (ZO-2 KD + ZO-2 re-expressing). (**A**) Schematic representation of the indentation setup. (**B**) Representative force-indentation curves fitted with Sneddon’s model for conical cantilever tips. (**C**) Stiffness or Young’s modulus of the apical surface of MDCK cells expressed in kPa. Each dot plot in the graph represents one value of Young’s modulus obtained from one force-indentation curve. Results derived from at least two independent experiments. n = number of fitted force-indentation curves used to generate this figure. Statistical analysis was conducted using Kruskal–Wallis one-way ANOVA and Dunn’s multiple comparisons tests. Horizontal lines correspond to medians. **** *p* < 0.0001. The dotted line in the ZO-2 KD + ZO-2 re-expressing group indicates where the data were divided into two groups to obtain the corresponding medians. (**D**) Schematic summary of results. The diminished apical rigidity observed in ZO-2 KD cells relates to the junctional dissociation of apical microtubules.

Stiffness or rigidity in Young’s module values is the extent to which a material resists deformation in response to an applied force (for review, see [19]). To test if ZO-2 regulates the mechanical tension of the apical membrane of epithelial cells, we measured the elastic Young’s modulus of the surface of parental and ZO-2 KD MDCK cells using nanoindentation technique with an atomic force microscope, based on the idea that the force which restores deformation originates from the tension of the apical membrane. For this purpose, parental and ZO-2 KD cells plated on glass slides were subjected to nanoindentation, as illustrated in the diagram of Figure 1A. Figure 1B shows representative force-indentation curves, fitted with Sneddon’s model for conical cantilever tips of parental or ZO-2 KD MDCK monolayers incubated with docetaxel for microtubule stabilization, with or without blebbistatin (a cell-permeable inhibitor of NMII [20]), or in a low calcium (LC) condition. The stiffness or Young’s modulus of the apical surface is lower in ZO-2 KD than in parental MDCK cells. It resembles that observed in monolayers incubated in LC (Figure 1C), where TJs disassemble [21]. In parental cells, treatment with blebbistatin diminishes the apical tension to values such as those present in ZO-2 KD cells and in monolayers incubated in the LC condition. 

Since others have found a slight increase in apical stiffness in either ZO-1 [11], ZO-2, or both ZO-1 and ZO-2 depleted MDCK cells [12], we decided to confirm further that the decrease in apical tension here observed was due to the lack of ZO-2. For this purpose, we measured the Young’s modulus of the surface of monolayers of ZO-2 KD cells that had not received a periodical zeocin treatment and hence had begun to re-express ZO-2 in cells scattered throughout the monolayer (ZO-2 KD + ZO-2 re-expressing). In these monolayers, we observed that the values of apical tension could be divided into two groups (Figure 1C, dotted line): one with a median resembling the one found in parental cells and the other with a median like that of ZO-2 KD MDCK cells. These results indicate that the presence of ZO-2 is critical to maintaining the tension of the apical membrane of MDCK cells.

A decrease in apical rigidity such as that found in ZO-2 KD MDCK cells was seen upon cingulin depletion [22]. In this respect, it was previously reported that the non-centrosomal planar apical microtubule network associates to the TJ through AMPK phosphorylated cingulin [23]. Previously, we found that ZO-2 favored AMPK-mediated cingulin phosphorylation and observed accordingly that the apical microtubule network was absent in ZO-2 KD cells [6]. These precedents prompted us to test if the apical tension of ZO-2 KD cells could be restored upon microtubule stabilization. For this purpose, we used docetaxel, a microtubule stabilizer (for review see [24]) that induces the appearance of thick bundles of microtubules around the cell borders in epithelial cells [25,26]. Figure 1C shows that a 4 h treatment with 10 μM docetaxel restored apical tension in ZO-2 KD cells, suggesting that the reduced apical rigidity observed in ZO-2 KD was related to the dissociation of apical microtubules from the TJ (Figure 1D).

### 2.2. The Lack of ZO-2 Reduces γ-Actin Concentration at the TJ and the Lateral Membrane, Promoting Its Accumulation at Stress Fibers Instead

The decrease in apical rigidity observed in epithelial cells upon cingulin depletion is accompanied by a diminished interaction of ZO-2 with γ-actin and a reduced accumulation of nonmuscle myosin 2B (NMIIB) at the TJ [22]. Hence, we investigated if the lack of ZO-2 altered the distribution of NMIIB. We observed that NMIIB in MDCK cells is present immediately below the TJ region at the AJ level and that the lack of ZO-2 has no impact on NMIIB distribution at the cell borders (Supplementary Figure S1).

**Figure 2:**
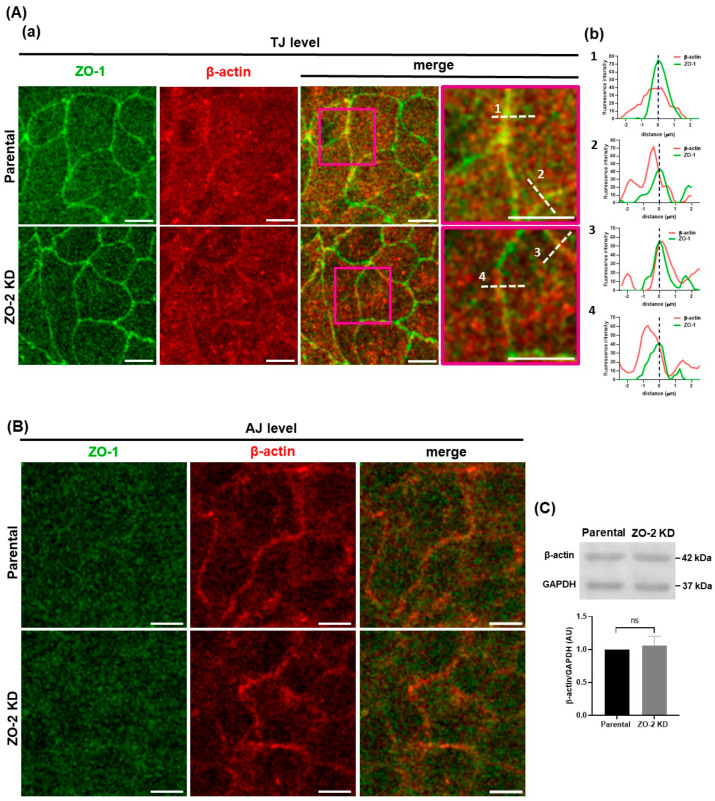

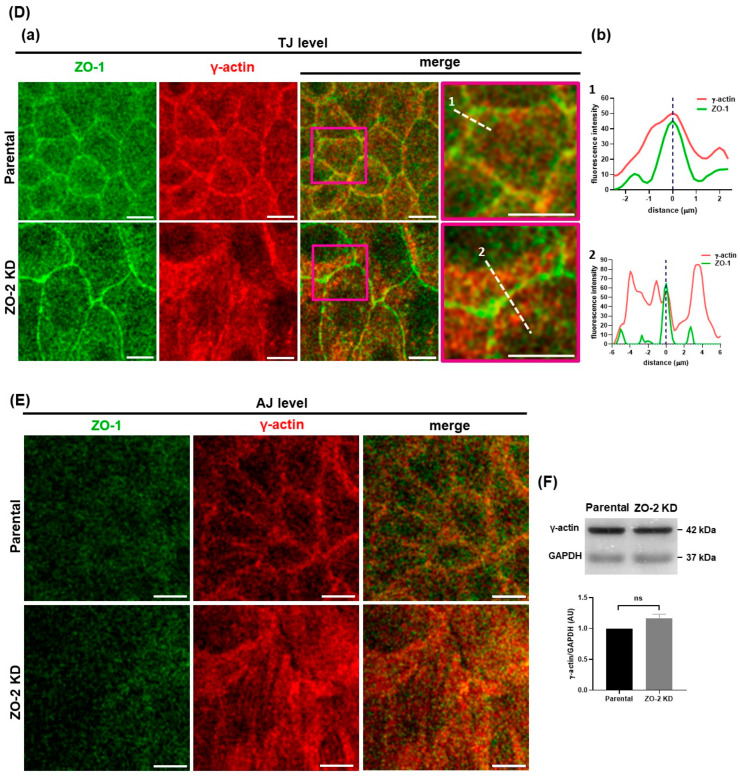

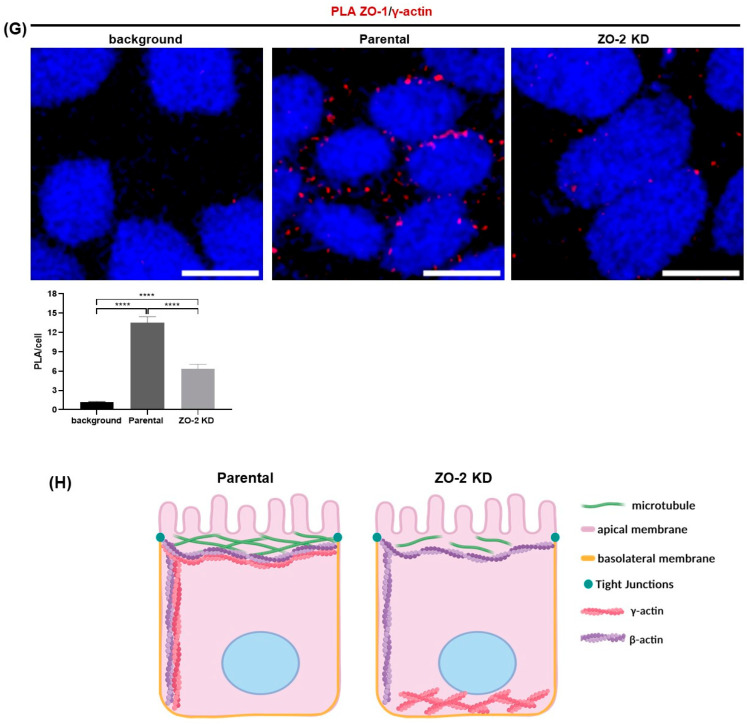
The lack of ZO-2 does not affect β-actin localization in MDCK cells but diminishes γ-actin interaction with the TJ, giving rise to a diffuse and broader staining pattern of γ-actin at the TJ level and to the proliferation of basal stress fibers. Parental and ZO-2 KD MDCK cells were processed for immunofluorescence with antibodies against ZO-1 and β-actin (**A**,**B**), or ZO-1 and γ-actin (**D**,**E**). (**Aa**,**Da**) Pile-up of optical fields taken at the TJ level (**A**,**D**), or along the lateral membrane, from the base of the cells up to the region immediately below the TJ (**B**,**E**). Images are representative of 6 optical fields analyzed per experimental condition, derived from two independent experiments. Scale bars, 10 µm. Pink squares in Aa and Da correspond to the areas amplified in the images shown at right with a pink frame, and white dashed lines indicate the location where the linescan analysis was done. The numbers in the linescans in Ab and Db correspond to the analysis done of the region indicated with a white dashedline and identified with a number in Aa and Da. (**Ab**,**Db**) Linescan analysis with fluorescence intensities of the two channels as a function of distance from the midline ZO-1 labeling indicated with a blue dashed line. (**C**) Western blot of β-actin in parental and ZO-2 KD cells. GAPDH was employed as a loading control. Upper panel, representative image; lower panel, quantitative analysis derived from three independent experiments. Statistical analysis was conducted with Student’s t-test. Results are shown as media ± standard deviation, ns, non-significant. (**F**) Western blot of γ-actin in parental and ZO-2 KD cells. GAPDH was employed as a loading control. Upper panel, representative image; lower panel, quantitative analysis derived from three independent experiments. Statistical analysis was conducted with Student’s *t*-test. Results shown as media ± standard deviation, ns, non-significant. (**G**) The lack of ZO-2 diminishes the interaction of γ-actin with ZO-1. PLA was carried out in parental and ZO-2 KD MDCK cells treated with mouse antibodies against γ-actin and rabbit antibodies against ZO-1. Red fluorescent spots reveal by PLA a positive interaction between ZO-1 and γ-actin. Background, PLA conducted in parental monolayers only with antibodies against ZO-1. Nuclei stained with DAPI. Bar, 10 µm. Top panel, representative images; bottom panel, quantitative analysis performed using BlobFinder version v3.2. Statistical analysis was conducted with a one-way analysis of variance (ANOVA) followed by the Tukey multiple comparison test. Results were obtained from six optical fields in each experimental condition that were derived from two independent experiments. **** *p* < 0.0001. (**H**) Schematic summary of results. ZO-2 depletion inhibits γ-actin association with TJs and instead triggers the formation of basal stress fibers of γ-actin.

Next, we analyzed if the lack of ZO-2 altered the distribution of β- and γ-actin, which in epithelial cells segregate under the basolateral and apical membranes, respectively [27]. In both parental and ZO-2 KD cells, β-actin at the TJ level displays a diffuse cytoplasmic pattern with a discreet concentration at the cell borders (Figure 2Aa). The analysis of the intensity profiles across the junction shows that in both parental and ZO-2 KD cells, there are regions at the cell border where the peaks for the signals for β-actin and ZO-1 colocalize (Figure 2Ab1,b3) and others where they do not (Figure 2Ab2,b4). β-actin present below the TJ concentrates at the lateral membrane, and the absence of ZO-2 does not change this pattern (Figure 2B). In Western blot, no difference in the amount of β-actin was observed between parental and ZO-2 KD MDCK cells (Figure 2C).

γ-Actin in epithelial cells is present as an apical pool of stable perijunctional bundles [27] and binds to ZO-2 at the cell periphery [22]. In Figure 2Da, ZO-2 KD cells display a broader and less concentrated distribution of γ-actin along the cell borders at the TJ level in comparison to parental cells, even when ZO-1 is concentrated at the TJ. The diffuse peripheral distribution of γ-actin was confirmed by the intensity profiles across the junction (Figure 2Db). In the lateral membrane, below the TJ region, γ-actin forms stress fibers in cells that lack ZO-2, whereas in parental cells, it concentrates at the lateral membrane (Figure 2E). Using Western blot, we detected no change in the amount of γ-actin between parental and ZO-2 KD MDCK cells (Figure 2F).

To further analyze the effect induced by the lack of ZO-2 on γ-actin concentration at the TJ region, we performed a proximity ligation assay (PLA) with antibodies against ZO-1 and γ-actin in parental and ZO-2 KD cells. Figure 2G reveals that the lack of ZO-2 diminishes the interaction of γ-actin with the TJ protein ZO-1. These observations suggest that the presence of ZO-2 at the TJ tethers γ-actin to the cell borders, facilitating in turn its interaction with junctional ZO-1.

Taken together, our results suggest that the diminished apical tension found in ZO-2 KD cells might be due to the reduced interaction of the apical pool of γ-actin and cortical microtubules with the TJ.

### 2.3. ZO-2 Silencing Increases the Mechanical Force at the TJ

**Figure 3:**
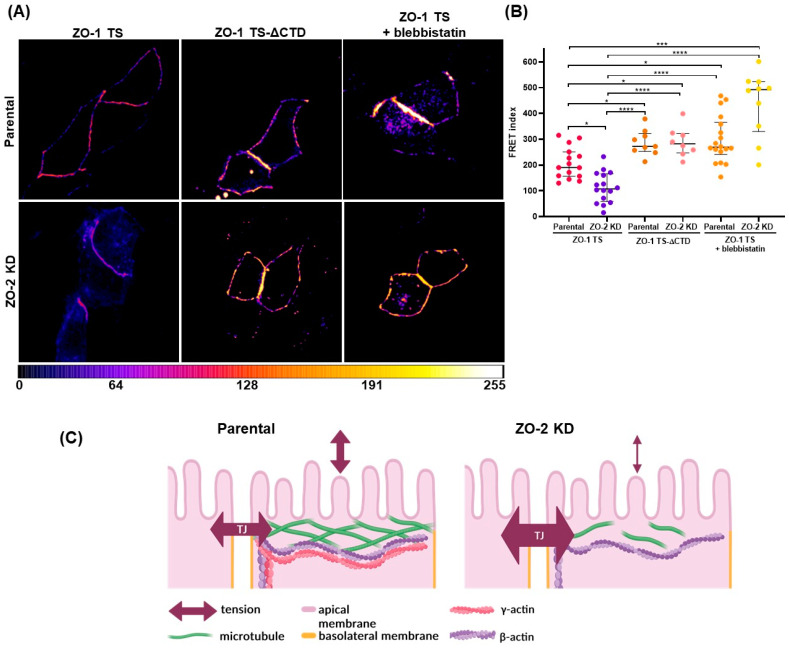
The lack of ZO-2 augments tension at the TJ. Parental and ZO-2 KD MDCK cells were transfected with ZO-1 TS or ZO-1 TS-ΔCTD. After 6 h, monolayers transfected with ZO-1 TS were treated or not with blebbistatin 100 µM for 15 h. (**A**) Representative FRET images of parental and ZO-2 KD MDCK cells are color-coded, where low to high FRET is in a scale of cold to warm colors. (**B**) Calculated FRET index of junctional segments in parental and ZO-2 KD cells with the indicated transfected FRET construct and treatment. Data was obtained from three independent experiments. Statistical analysis was performed with Kruskal–Wallis one-way ANOVA and uncorrected Dunn’s multiple comparisons tests. Horizontal lines correspond to the median and interquartile range. **** *p* < 0.0001, *** *p* < 0.001, * *p* < 0.05. (**C**) Schematic summary of results. ZO-2 depletion increases mechanical tension at the TJ.

Next, we sought to investigate if the absence of ZO-2 triggered a change in the mechanical tension at the TJ. To measure this, we employed a ZO-1 tension sensor (ZO-1 TS) in which the elastic FRET module was inserted after the MAGUK segment of the molecule containing the three PDZ domains, the SH3 and GuK modules, and the carboxy-terminal domain with the α alternative segment, the actin-binding region (ABR), and the ZU5 domain [15]. This sensor was chosen because previous experiments had shown that it is efficiently expressed at TJs and responds with increased FRET efficiency upon relaxation due to myosin inhibition with blebbistatin [15]. Figure 3 reveals that the FRET index of the full-length ZO-1 sensor is lower in ZO-2 KD than in parental MDCK cells, indicating that tension at the TJ is higher in the absence of ZO-2. The maximal FRET index and, thus, the lower tension level is observed when parental and ZO-2 KD cells are treated with blebbistatin 100 µM or the experiment is conducted with a FRET construct that, due to the lack of the carboxy-terminal domain of ZO-1 (ZO-1 TS-ΔCTD), cannot sense tensile stress.

### 2.4. In ZO-2 KD MDCK Cells, the Expression of JAM-A at the Cell Border Diminishes while That of p114RhoGEF Augments

**Figure 4:**
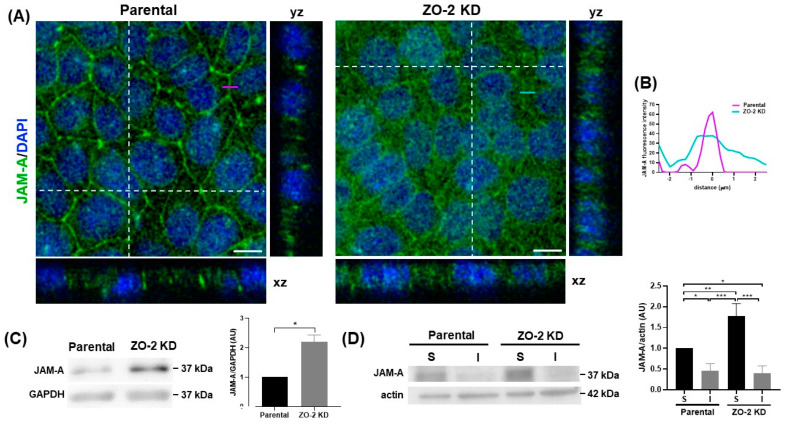
JAM-A expression at the cell borders diminishes in ZO-2 KD cells. (**A**) Monolayers of parental and ZO-2 KD MDCK cells were processed for immunofluorescence with antibodies against JAM-A. Nuclei were stained with DAPI. Scale bars, 10 µm. Representative images of pile-ups and confocal sections in xz and yz planes made along the white dotted lines, obtained with ImageJ version 1.54f. Two independent experiments were conducted, and three optical fields per condition were analyzed in each experiment. Purple and green lines indicate the site where the line scan analysis shown in B was done. (**B**) Line scan analysis of purple and green lines in A show fluorescence intensity as a function of distance from the midline JAM-A labeling. (**C**) Western blot analysis of JAM-A in parental and ZO-2 KD MDCK cells. GAPDH was employed as a loading control. Left, representative image; right, quantitative analysis. Statistical analysis was conducted with Student’s *t*-test. Results are shown as media ± standard deviation, * *p* < 0.05. (**D**) Western blot analysis of JAM-A present in soluble (S) and insoluble (I) fractions was conducted in parental and ZO-2 KD MDCK cells. Actin was employed as a loading control. Left: a representative image of three independent experiments; right: a quantitative analysis. Statistical analysis was conducted with one-way ANOVA followed by Tukey’s multiple comparisons test. Results are shown as media ± standard deviation. * *p* < 0.05, ** *p* < 0.01, *** *p* < 0.001.

The increase in the tensile stress of the ZO-1 sensor observed upon ZO-2 depletion is akin to that found when JAM-A is silenced [15]. This caught our attention, since JAM-A depletion triggers in MDCK cells profound mechanical alterations, including myosin light chain phosphorylation along stress fibers and at cell-cell junctions, as well as abundant stress fibers [15]. Hence, we next tested if the expression of JAM-A is altered upon ZO-2 silencing. Figure 4A shows via immunofluorescence that, in comparison to parental MDCK cells, in ZO-2 KD cells, JAM-A is less concentrated at the cell borders and instead exhibits a conspicuous diffuse cytoplasmic distribution, as confirmed by the analysis of intensity profiles across the junction (Figure 4B). Through Western blot, we detected an increase in the total content of JAM-A in ZO-2 KD cells (Figure 4C). This apparent contradictory result was explained during the analysis of soluble and insoluble fractions, where we found that ZO-2 KD cells had a significantly higher amount of soluble JAM-A than parental cells (Figure 4D). 

**Figure 5:**
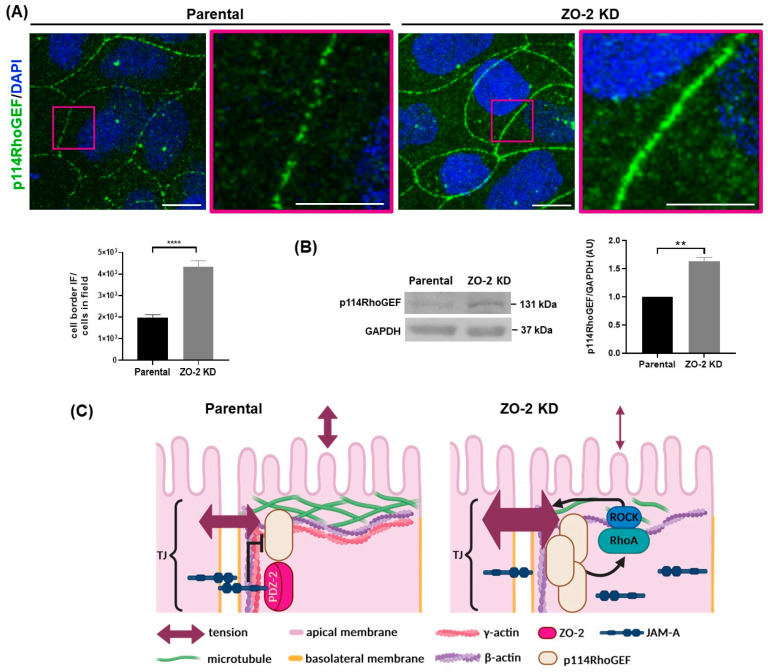
The expression of p114RhoGEF increases in ZO-2 KD cells. (**A**) Monolayers of parental and ZO-2 KD MDCK cells were processed for immunofluorescence with antibodies against p114RhoGEF. Pink squares indicate the areas amplified in the images shown at right with a pink frame. Nuclei were stained with DAPI. Upper panels, representative images; Scale bars, 10 µm; lower left panel, fluorescence quantitative analysis of pile-up images, conducted with ImageJ version 1.54f. Data were obtained from 16 optical fields per experimental condition and two independent experiments. Statistical analysis was carried out using Student’s *t*-test. Results are shown as media ± standard deviation. **** *p* < 0.0001. (**B**) Western blot analysis of p114RhoGEF in parental and ZO-2 KD MDCK cells. GAPDH was employed as a loading control. Left, representative images; right, quantitative analysis. Statistical analysis was conducted using Student’s *t*-test. Results are shown as media ± standard deviation, ** *p* < 0.001. (**C**) Schematic summary of results. The lack of ZO-2 inhibits JAM-A association to TJs and allows p114RhoGEF junctional concentration, which triggers actomyosin contraction enhancing tension at the TJ.

NMII activation in JAM-A depleted cells is attenuated by p114RhoGEF silencing, indicating that actomyosin regulation downstream of JAM-A requires p114RhoGEF, which triggers RhoA and ROCK activation [15]. Moreover, the increase in stress of the ZO-1 sensor generated upon JAM-A silencing was explained by the blockade of p114RhoGEF activity exerted by JAM-A [15]. Therefore, we next analyzed the impact of ZO-2 silencing on p114RhoGEF. Figure 5A shows that in the absence of ZO-2, the pattern of p114RhoGEF at the cell border is more continuous and intense than that observed in parental MDCK cells. Correspondingly, a Western blot analysis reveals a higher cellular content of p114RhoGEF in ZO-2 KD cells in comparison to parental MDCK (Figure 5B). Altogether, these observations indicate that the absence of ZO-2 inhibits JAM-A association to the cell borders, facilitating p114RhoGEF junctional concentration that leads to an enhanced tension at the TJ level (Figure 5C). 

### 2.5. In Silico Analysis Shows That JAM-A Binds to ZO-2 PDZ-2 Domain via Stable Hydrophobic Interactions

**Figure 6:**
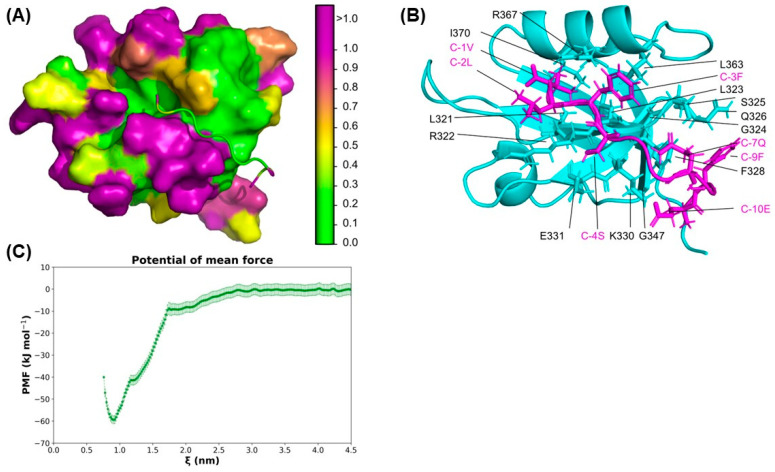
In silico analysis of JAM-A tail and ZO-2 PDZ-2 complex. (**A**) PARCH scale calculations were performed for the ZO-2 PDZ-2 domain in complex with the last ten residues (EFKQTSSFLV) of JAM-A’s C-terminal tail. The complex is shown using surface representation for the ZO-2 PDZ-2 domain and cartoon representation for the JAM-A. The residues are colored based on the parch values on the color scale (right). The PDZ-2 domain forms a long hydrophobic pocket facilitating the binding of the JAM-A tail’s FLV residues. (**B**) Key residues of the JAM-A tail (magenta) and ZO-2 PDZ-2 (cyan) are shown in cartoon and stick representation. (**C**) The potential of mean force curve for unbinding JAM-A tail from ZO-2 PDZ-2 domain. Error bars were calculated via the Bayesian bootstrapping method using the GROMACS WHAM option. The binding energy of the JAM-A tail and ZO-2 PDZ-2 complex is −59.4 ± 1.75 kJ/mol.

Since the lack of ZO-2 diminishes JAM-A concentration at the cell borders, we next analyzed in silico the interaction between the C-terminal tail of JAM-A and the PDZ-2 domain of ZO-2. The hydropathy of the PDZ-2/JAM-A complex was assessed using PARCH scale calculations [28]. Based on its chemical and topographic affinity for water, each amino acid residue was assigned a parch value on a scale from 0 to 10, where low parch values (<1) indicate hydrophobic character. The PARCH method provides a quantitative assessment of protein–water interactions based on the chemical and topographical nature of the protein. Other hydropathy assessment methods overlook the nanoscale topography of the protein and assign fixed value values to the 20 amino acids to classify them as hydrophilic or hydrophobic. In the PDZ2/JAM-A complex, the role of topography is critical in assessing the stability of interactions. We found that JAM-A’s last seven residues, QTSSFLV, are hydrophobic, while the remaining three EFK residues of the 10-mer are hydrophilic. Interestingly, the last residue of the JAM-A tail, valine, has a slightly higher PARCH value than the other six of the hydrophobic side of the tail. We infer this is because of the COO- being present and surrounding hydrophilic glutamic acid residues just outside the hydrophobic pocket. The JAM-A tail is stabilized by the hydrophobic pocket formed by ZO-2 PDZ-2 residues from Y319 to V329 and I370 (Figure 6).

The pair-wise residue contacts also provided insight into the interactions between the PDZ-2 domain and the JAM-A 10-mer. Out of the ten JAM-A residues, seven residues made contact (Table 1). Most residues make multiple contacts in an overall topologically hydrophobic PDZ-2 pocket. The binding energy of JAM-A with ZO-2 PDZ-2 is −59.4 ± 1.75 kJ/mol. The minima in the free energy curve at the intermolecular distance, ξ = 0.9 nm correspond to the binding of the FLV residues during the steered dynamics simulations. 

### 2.6. The Expression of Afadin Augments in ZO-2 KD MDCK Cells

**Figure 7:**
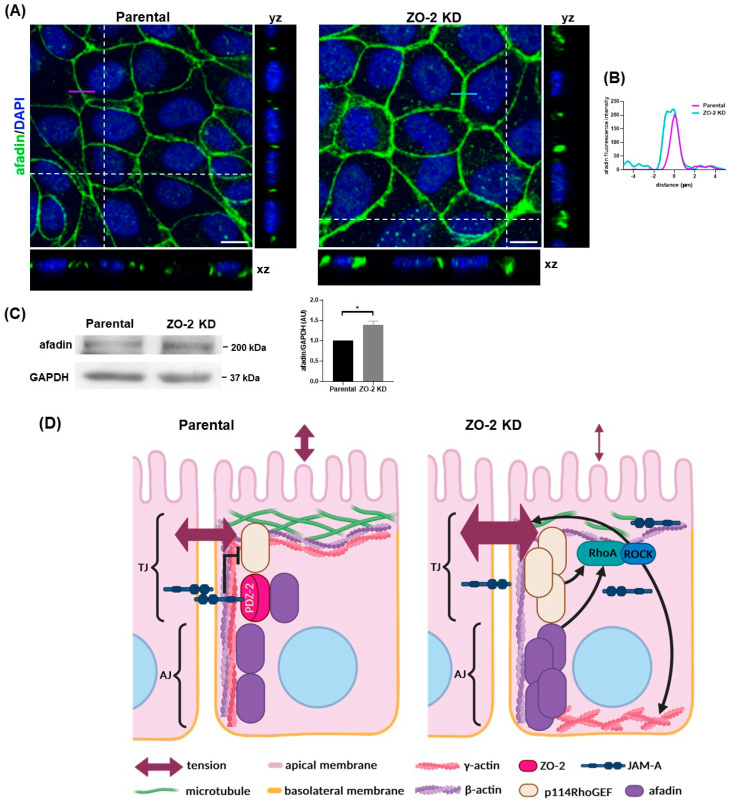
The expression of afadin increases in ZO-2 KD cells. (**A**) Monolayers of parental and ZO-2 KD MDCK cells were processed for immunofluorescence with antibodies against afadin. Nuclei were stained with DAPI. (**A**) Left, representative images of pile-ups and confocal sections in xz and yz planes made along the white dotted lines, obtained with ImageJ version 1.54f. Purple and green lines indicate the place where the line scan analysis shown in B was done. Three independent experiments were conducted, and three optical fields per condition were analyzed in each experiment. Scale bars, 10 µm. (**B**) Line scan analysis of purple and green lines in A show fluorescence intensity as a function of distance from the midline afadin labeling. (**C**) Western blot analysis of afadin in parental and ZO-2 KD MDCK cells. GAPDH was employed as a loading control. Left, representative image; right, quantitative analysis. Statistical analysis was conducted with Student’s *t*-test. Results are shown as media ± standard deviation, * *p* < 0.05. (**D**) Schematic summary of results. In ZO-2 KD cells, afadin is recruited to the cell borders, and through the activation of Rho/ROCK the tension at the TJ augments and abundant basal stress fibers are formed.

ZO-2 associates with afadin, an actin filament-binding protein [29], and together with JAM-A these proteins form a complex where the interaction of afadin with JAM-A depends on ZO-2 expression [7]. Afadin is required for the RhoA/ROCK-dependent formation of actin stress fibers, and its depletion inactivates RhoA and reduces NMII phosphorylation [30]. In addition, junctional afadin has been shown to augment in response to elevated contractility in ZO-1 and ZO-2 double KD cells [10]. Therefore, we next investigated if the expression of afadin changed in cells that only lack ZO-2. Figure 7A shows, via immunofluorescence, a wider band of afadin at the cell borders of ZO-2 KD cells compared to that observed in parental MDCK cells. The analysis of the afadin intensity profile across the junction confirmed that a wider afadin band was present in ZO-2 KD cells (Figure 7B). The Western blot analysis also revealed a higher concentration of afadin in ZO-2 KD cells (Figure 7C). 

Altogether, these results indicate that in the absence of ZO-2, JAM-A association with the cell border diminishes while the junctional recruitment of afadin and p114RhoGEF augments, increasing mechanical tension at the TJ and the appearance of basal stress fibers (Figure 7D).

### 2.7. The Absence of ZO-2 Exerts No Effect on Tension at the Adherens Junction

**Figure 8:**
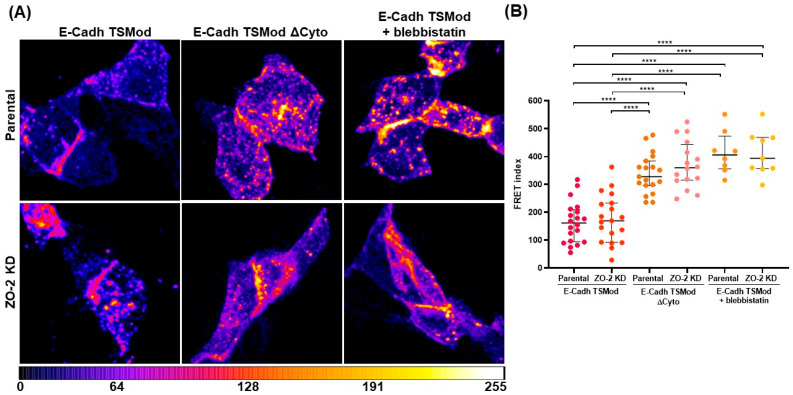
The lack of ZO-2 does not affect tension at the adherens junction. Parental and ZO-2 KD MDCK cells were transfected with E-cad TSMod or E-cad TSModΔcyto. After 6 h, monolayers transfected with E-cad TSMod were treated or not with blebbistatin 100 µM for 15 h. (**A**) Representative FRET images of parental and ZO-2 KD MDCK cells are color-coded, where low to high FRET is on a scale from cold to warm colors. (**B**) Calculated FRET index of junctional segments in parental and ZO-2 KD cells with the indicated transfected FRET construct and treatment. Data were obtained from three independent experiments. Statistical analysis was conducted with Kruskal–Wallis one-way ANOVA and uncorrected Dunn’s multiple comparisons tests. Horizontal lines correspond to the median and interquartile range. **** *p* < 0.0001.

Next, we investigated if the absence of ZO-2 altered the mechanical tension at the AJ. For this purpose, parental and ZO-2 KD cells were transfected with the E-cadherin tension sensor (E-Cadh TSMod) containing between its transmembrane domain and the β-catenin binding domain a tension module (TSMod) with TFP and YFP fluorophores separated by an elastic linker, as previously described [31]. Figure 8 shows that the FRET index in ZO-2 KD MDCK cells is no different than that of parental cells, thus indicating that the absence of ZO-2 exerts no effect on tension mediated by E-cadherin. As further controls, to achieve maximum FRET that corresponds to minimal mechanical tension, additional FRET measurements were carried out with parental and ZO-2 KD cells transfected with E-Cadh TSMod and treated for 15 h with blebbistatin 100 µM or with cells transfected with E-cad TSModΔcyto. Since the β-catenin binding domain was missing, this construct could not sense tension. As expected, maximum FRET was observed in the latter conditions, and no difference was found between parental and ZO-2 KD cells.

### 2.8. The Lack of ZO-2 Induces the Recruitment of Vinculin to Tricellular TJs, Indicative of Higher Mechanical Tension at These Junctions

**Figure 9:**
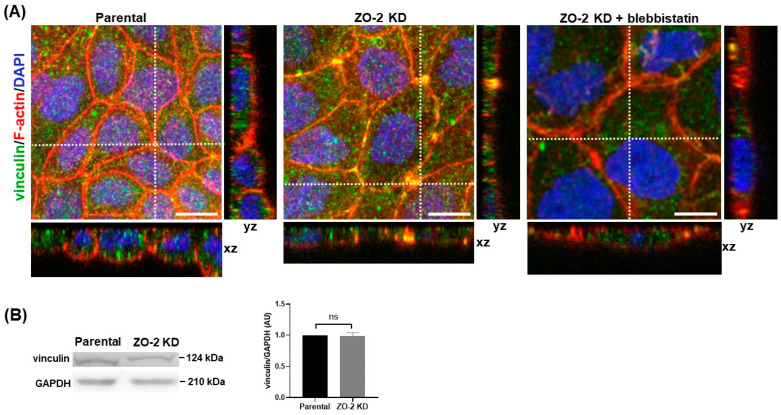
Vinculin concentrates at tricellular and multicellular junctions in ZO-2 KD cells. (**A**) Monolayers of parental and ZO-2 KD MDCK cells treated or not with blebbistatin 100 µM for 15 h were processed for immunofluorescence with antibodies against vinculin (green) and rhodaminated phalloidin to detect F-actin (red). Nuclei were stained with DAPI. Representative images of pile-ups and confocal sections in xz and yz planes made along the white dotted lines, obtained with ImageJ version 1.54f. Two independent experiments were conducted, and three optical fields per condition were analyzed in each experiment. Scale bars, 10 µm. (**B**) Western blot analysis of vinculin in parental and ZO-2 KD MDCK cells. GAPDH was employed as a loading control. Left, representative image; right, quantitative analysis. Statistical analysis was conducted with Student’s *t*-test. Results are shown as media ± standard deviation; ns, non-significant.

Although the E-cadherin tension sensor revealed no increase in lateral tension in ZO-2 KD cells compared to parental cells, we investigated if a change in tension was present in ZO-2 KD cells at tTJs. The latter are specialized TJs that obliterate the extracellular space at tricellular or multicellular contacts and are constituted of the most apical elements of TJ strands in bicellular TJs that join at tricellular contacts, turn downwards, and extend in the basal direction, forming central sealing elements that squeeze the extracellular space and hence work as a diffusion barrier [32]. At tTJs, tricellulin is known to bind directly to α-catenin, enabling tension-dependent vinculin recruitment [33]. Therefore, we next analyzed if the expression of vinculin at tTJs was altered in ZO-2 KD MDCK cells. Figure 9A reveals that the absence of ZO-2 is sufficient to induce the appearance of vinculin at the cellular borders and its conspicuous concentration at tricellular and multicellular junctions. 

Exposure of the vinculin-binding region of α-catenin requires stretching the α-catenin molecule by mechanical forces applied to its C terminus, derived from actomyosin contraction [34,35]. Therefore, we next examined if inhibiting myosin II activity with blebbistatin blocked vinculin recruitment to tTJs. Our results indicate that treatment of ZO-2 KD MDCK cells with blebbistatin 100 µM abolishes vinculin concentration at tTJs (Figure 9A). These observations suggest that in the absence of ZO-2, a high mechanical force is applied at tTJs.

Western blot analysis shows no difference in the expression level of vinculin between ZO-2 KD cells and parental MDCK cells (Figure 9B). A similar situation was previously found among Eph4 wild-type cells and tricellulin KO cells [36], thus indicating that changes in vinculin recruitment do not alter the expression level of the protein. 

Taken together, our results indicate that the lack of ZO-2 in MDCK cells increases junctional tension at tTjs by actomyosin contraction, triggering α-catenin stretching and vinculin recruitment to tTJs.

### 2.9. Increased Tension Allows ZO-2 KD Monolayers to Form Holes When Plated on 20 kPa Hydrogels

**Figure 10:**
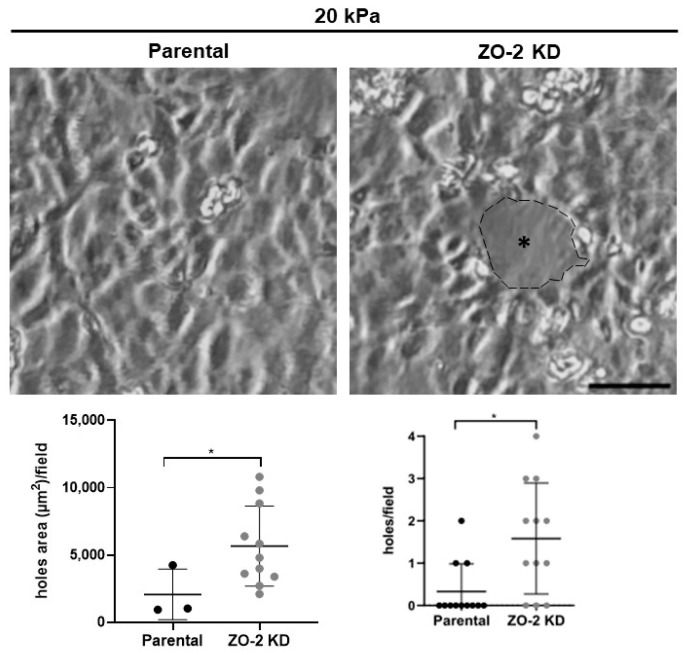
The lack of ZO-2 allows hole formation in MDCK monolayers cultured in 20 kPa hydrogels. Parental and ZO-2 KD MDCK monolayers were plated at confluence density on hydrogels with 20 kPa rigidity, covered with fibronectin. Hole formation was analyzed 24 h later. Upper panel, representative images; bar, 50 μm. *, hole outlined with a discontinuous line. Lower panel, quantification of the number of holes per field, and the area covered by the holes per field. Results were obtained from at least two independent experiments, and 12 optical fields were analyzed per condition. Statistical analysis was conducted with Mann–Whitney test. Results are shown as media ± standard deviation, * *p* < 0.05.

A recent report indicated that a build-up of junctional tension leads to spontaneous hole formation in monolayers plated on soft 2.3–8.6 kPa hydrogels [37]. Here, we have observed in ZO-2 KD cells an increase in tension at the TJ measured with the ZO-1 tension sensor and at tTJs detected by vinculin recruitment. Hence, we next analyzed if the increased junctional tension detected in ZO-2 KD cells could trigger hole formation in monolayers plated on hydrogels with 20 kPa rigidity covered with fibronectin. This rigidity was chosen because MDCK monolayers do not develop holes when plated on hydrogels with rigidities between 16 and 55 kPa [37]. Figure 10 shows that the lack of ZO-2 triggers the formation of holes in MDCK monolayers, while a negligible amount was detected in parental cells. These results thus confirm that ZO-2 KD cells are in a high junctional tensile state.

### 2.10. The Absence of ZO-2 Retards the Elongation of Cells Plated in Collagen IV or Fibronectin, and the Formation of Cell Aggregates in Soft and Stiff Substrates

The stiffness of the ECM is a parameter that significantly impacts cell behavior (for review, see [38]), and the translation of the stiffness of the ECM into cell responses is mediated by heterodimers of integrins (for review, see [39]). Integrins associated with their ECM ligands form clusters that trigger the assembly of focal adhesions between the cell and the ECM. Cells grown on stiff matrices compared to soft ones increase cell spreading, augment the number of focal adhesions [16,17], and display increased ROCK activation, leading to actin cytoskeleton contractility, FAK activation, and stress fibers formation [16,40]. In ZO-2 KD cells we previously found a diminished expression of integrin β1, an overactivity of RhoA/ROCK, and abundant stress fibers [6], whose presence can now be explained by the junctional accumulation of p114RhoGEF, which, as previously reported, triggers RhoA/ROCK activation [41]. 

These observations prompted us to analyze the spreading and aggregation of parental and ZO-2 KD cells plated on hydrogels with 1 or 20 kPa rigidity covered with collagen IV or fibronectin. We studied cell aggregation, since ZO-2 is a TJ protein whose absence is expected to reduce cell–cell contact development; and we analyzed cell spreading because it is the result of a balance between cell–cell and cell to substratum adhesion [42], and in ZO-2 KD cells we observe abundant stress fibers [6]. 

**Figure 11:**
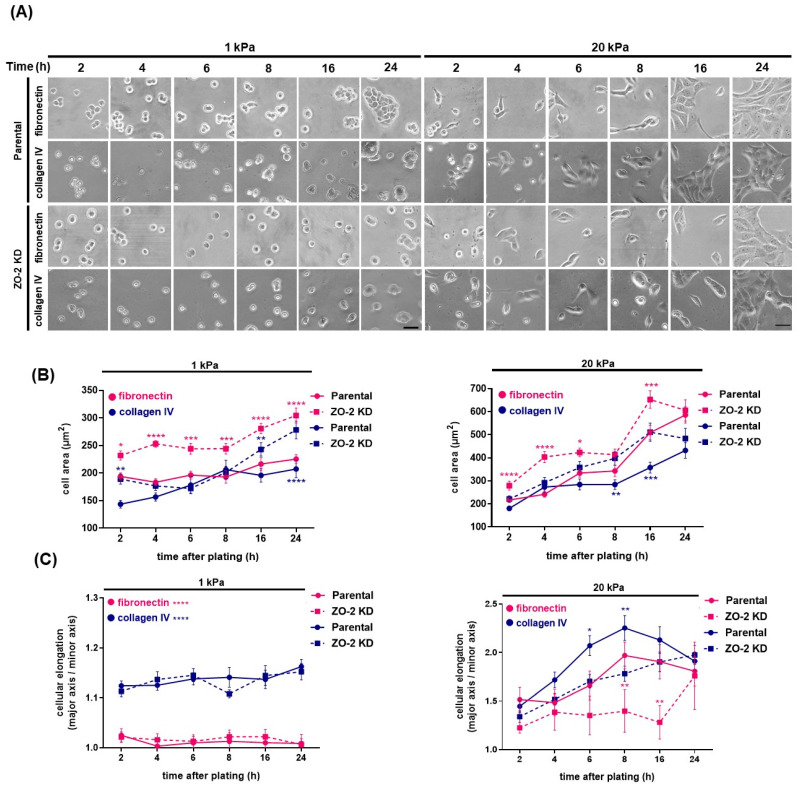

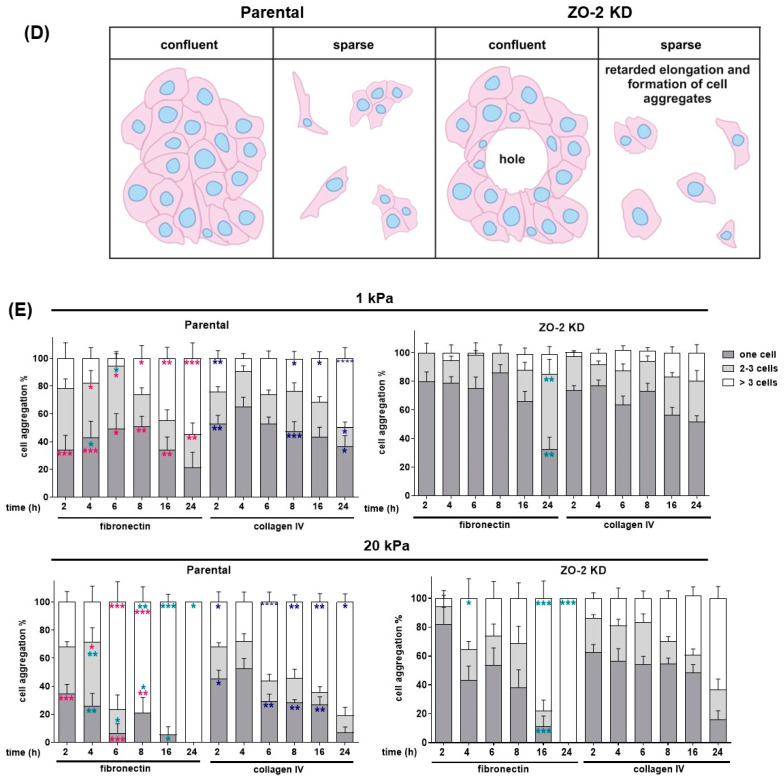
The lack of ZO-2 slows down the elongation of cells plated in stiff substrates covered with collagen IV or fibronectin and diminishes cell aggregation in soft and stiff substrates. (**A**) Light microscopy images of parental and ZO-2 KD MDCK cells at different times after plating on Ac/Bis Ac hydrogels with either 1 or 20 kPa rigidity, covered with collagen IV or fibronectin. Representative images of three independent experiments. Bar, 30 μm. (**B**) Quantification of cell surface area at different times after plating. Statistical analysis was conducted with two-way ANOVA followed by Fisher’s LSD multiple comparisons test. From 27 to 60 cells were analyzed per condition and time. Results from three independent experiments are shown as media ± standard error. * *p* < 0.05; ** *p* < 0.01; *** *p* < 0.001; **** *p* < 0.0001 comparing ZO-2 KD vs. parental cells cultured on fibronectin (pink) or collagen IV (blue). (**C**) Quantification of cellular elongation at different times after plating. Statistical analysis was conducted with two-way ANOVA followed by Fisher’s LSD multiple comparisons test. From 27 to 60 cells were analyzed per condition and time. Results from three independent experiments are shown as media ± standard error. * *p* < 0.05; ** *p* < 0.01; **** *p* < 0.0001 comparing ZO-2 KD vs. parental cells cultured on fibronectin (pink) or collagen IV (blue). (**D**) Schematic summary of results. The lack of ZO-2 delays the elongation and reduces the aggregation of cells plated in 20 kPa hydrogels. (**E**) Quantification of cell aggregation at different times after plating. Statistical analysis was conducted with two-way ANOVA followed by Fisher’s LSD multiple comparisons test. From 27 to 60 cells were analyzed per condition and time. Results from three independent experiments are shown as media ± standard error. * *p* < 0.05; ** *p* < 0.01; *** *p* < 0.001; **** *p* < 0.0001; cyan *, fibronectin vs. collagen IV; pink *, parental vs. ZO-2 KD on fibronectin; blue *, parental vs. ZO-2 KD on collagen IV.

Figure 11A,B shows that ZO-2 KD cells display a higher surface area than parental cells, independently of the rigidity of the substrate or the nature of the extracellular matrix protein on which they were plated. These observations align with a previous observation showing that lacking ZO-2 triggers hypertrophy in MDCK cells [43]. 

MDCK cell elongation is severely restricted in cells plated on gels with 1 kPa rigidity, independent of the presence of ZO-2 or the nature of the extracellular matrix (ECM) protein on which they are seeded (Figure 11A,C). This observation agrees with a previous one showing that the spreading area of single cells on 0.6 kPa gels is six times smaller than on glass substrates [44]. 

Parental and ZO-2 KD cells plated on substrates with 20 kPa rigidity elongate with time after plating, and a substrate of collagen IV allows a higher elongation than fibronectin. The lack of ZO-2 retards cell elongation in both collagen IV and fibronectin (Figure 11D). However, this effect is more pronounced in the cells cultured on fibronectin than in collagen IV (Figure 11A,C). Cells cultured on hydrogels with 1 kPa rigidity, covered with fibronectin or collagen IV, do not elongate with time after plating (Figure 11A,C). We also analyzed cell aggregation with time after plating in parental and ZO-2 KD cells cultured in soft (1 kPa) and stiff (20 kPa) substrates covered with collagen IV and fibronectin. Figure 11A,E shows that both parental and ZO-2 KD cells display an increased cell aggregation with time after plating, which is more pronounced on the substrate with 20 kPa rigidity than on one with 1 kPa. In addition, cell aggregation is higher in the stiff substrate covered with fibronectin than in surfaces covered with collagen IV. However, the lack of ZO-2 delays the formation of cell aggregates in soft and stiff substrates, and in fibronectin and collagen IV-covered hydrogels (Figure 11A,D,E). 

### 2.11. In Cells Cultured in Nanostructured Ridge Arrays, the Lack of ZO-2 Accentuates the Cytoplasmic Accumulation of Claudin-4, While the Absence of ZO-2 Is Sufficient to Induce the Nuclear Concentration of Snail and YAP

Finally, we tested if the lack of ZO-2 affected the sensitivity of MDCK cells to the organization of the ECM. For this purpose, we plated parental and ZO-2 KD cells on nanostructured ridge arrays (NRA). The latter have nanoscale aligned fibers similar in size and organization to ECM fibers and are known to trigger partial and complete EMT-like processes [18].

**Figure 12:**
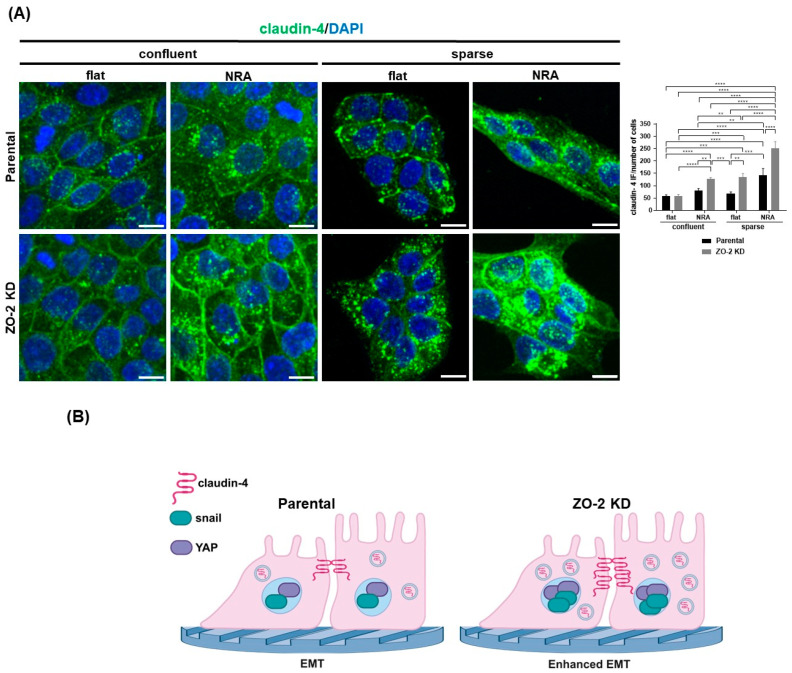

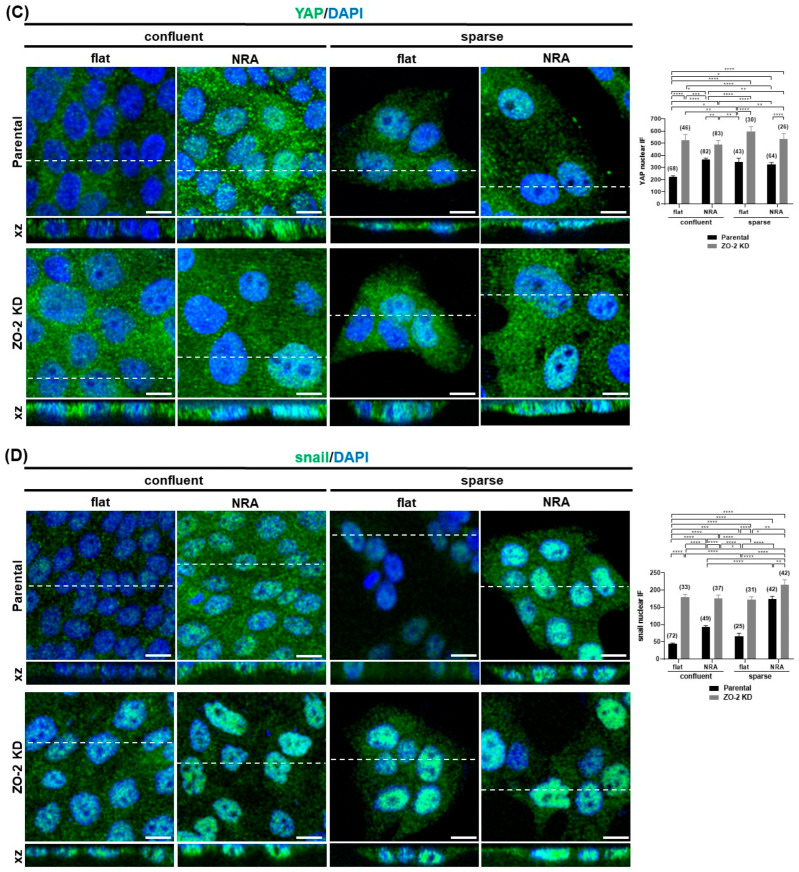
MDCK ZO-2 KD cells plated on NRA substrates accumulate claudin-4 in cytoplasmic vesicles, while the lack of ZO-2 is sufficient to induce YAP and Snail nuclear concentration. Parental and ZO-2 KD cells were plated at confluent (5 × 10^5^ cells/cm^2^) or sparse (3.7 × 10^4^ cells/cm^2^) density on glass coverslips or NRA substrates and analyzed 24 h later. (**A**) Monolayers were fixed and processed for immunofluorescence with antibodies against claudin-4. Nuclei were stained with DAPI. Left, representative images; bar, 10 µm; right, immunofluorescence quantification. Results were obtained from two independent experiments, where three optical fields per condition were analyzed in each experiment. Statistical analysis was conducted with two-way ANOVA followed by uncorrected Fisher’s LSD multiple comparisons test. Results shown as media ± SEM. ** *p* < 0.01; *** *p* < 0.001; **** *p* < 0.0001. (**B**) Schematic representation of results. ZO-2 KD cells plated on NRA substrates have a more intense accumulation of claudin-4 in cytoplasmic vesicles than parental cells. Although the sole absence of ZO-2 is sufficient to trigger YAP and Snail nuclear concentration, plating on NRA substrates enhances this effect. (**C**) Monolayers were fixed and processed for immunofluorescence with antibodies against YAP. Nuclei were stained with DAPI. Left, representative images of pile-ups and confocal sections in xz plane made along the white dotted line, obtained with ImageJ version 1.54f. Bar, 10 µm. Right, nuclear immunofluorescence quantification. Numbers in parentheses indicate the number of nuclei analyzed. Results were obtained from two independent experiments, where three optical fields per condition were analyzed in each experiment. Statistical analysis was conducted with two-way ANOVA followed by uncorrected Fisher’s LSD multiple comparisons test. Results shown as media ± standard error, * *p* < 0.05, ** *p* < 0.01; *** *p* < 0.001; **** *p* < 0.0001. (**D**) Monolayers were fixed and processed for immunofluorescence with antibodies against Snail. Nuclei were stained with DAPI. Left, representative images of pile-ups and confocal sections in xz plane made along the white dotted line, obtained with ImageJ version 1.54f. Bar, 10 µm. Right, nuclear immunofluorescence quantification. Numbers in parenthesis indicate the number of nuclei analyzed. Results were obtained from two independent experiments, where three optical fields per condition were analyzed in each experiment. Statistical analysis was conducted with two-way ANOVA followed by uncorrected Fisher’s LSD multiple comparisons test. Results shown as media ± standard error, * *p* < 0.05, ** *p* < 0.01; *** *p* < 0.001; **** *p* < 0.0001.

During EMT, epithelial cells lose their cell–cell adhesion properties, have decreased E-cadherin, and display an altered pattern of claudin expression (for review, see [18]). Hence, we analyzed if the absence of ZO-2 altered claudin-4 expression in cells cultured on NRA. We analyzed claudin-4, since previous reports had shown that it is more abundant in MDCK cells, stably expressing E7 oncoprotein from human papillomavirus 16 [45], and displays an altered pattern of expression in a wide variety of carcinomas (for review, see [46]). Figure 12A shows that 24 h after parental and ZO-2 KD cells were plated on NRA, in comparison to the flat surface of coverslips, claudin-4 expression in the cytoplasmic vesicles augmented, both in sparse and confluent cultures. However, in MDCK ZO-2 KD cells, this effect was accentuated. This result suggests that the lack of ZO-2 accentuates the recycling of claudin-4, triggered by the topography of the substrate (Figure 12B). 

Next, we analyzed in parental and ZO-2 KD cells cultured on NRA or glass coverslips for 24 h, the nuclear expression of the transcriptional activator YAP (Yes-associated protein), and the Snail transcription factor. We studied Snail because it is a prominent inducer of EMT (for review, see [47]), and YAP because when it is not phosphorylated by the hippo pathway kinase LATS, it travels to the nucleus, promoting the transcription of genes involved in EMT and the silencing of genes crucial for differentiation [18]. Figure 12B shows that the lack of ZO-2 is sufficient to induce the concentration of YAP at the nucleus, whereas in parental cells this phenotype is only present in sparse cultures and confluent monolayers cultured on NRA, as was previously shown [18,48]. 

Figure 12C shows that the lack of ZO-2 induces the nuclear accumulation of Snail and that this effect is more pronounced in cells grown on an NRA. This result indicates that the absence of ZO-2 accentuates the sensitivity of epithelial cells towards EMT triggered by nanoscale topography.

In summary, our results reveal that ZO-2 is a mechano-sensing protein that modulates tension at the apical membrane and the TJ, mediating the interaction of γ-actin with the TJ, favoring the recruitment of JAM-A to TJs and inhibiting the junctional accumulation of p114RhoGEF and afadin (Figure 7D). ZO-2 is also required by the cells to elicit an appropriate response to changes in the stiffness (Figure 11D) and topography of the substrate (Figure 12B). 

## 3. Discussion

Our atomic force microscopy experiments reveal that apical membrane rigidity diminishes in the absence of ZO-2 and can be restored in cells that re-express ZO-2 or upon microtubule stabilization with docetaxel. This decrease in apical rigidity is also obtained in parental cells treated with blebbistatin or incubated in the absence of extracellular calcium that triggers TJs opening [21]. This observation thus indicates that the presence of ZO-2 at the TJ is critical to maintaining apical stiffness through the interaction of the non-centrosomal planar apical microtubule network with the TJ.

A decreased apical rigidity accompanied by a diminished interaction of ZO-2 with γ-actin was observed upon cingulin depletion [22]. Hence, we investigated if the lack of ZO-2 altered the distribution of β- and γ-actin, which are associated with AJs and TJs, respectively [27]. We found that ZO-2 presence is necessary to maintain γ-actin closely associated with the TJ. Without ZO-2, γ-actin is redistributed from the lateral membrane to stress fibers. Therefore, the reduced apical rigidity observed in ZO-2 KD might be due to the dissociation from the TJ of both γ-actin and microtubules.

Our observation that γ-actin forms basal stress fibers in ZO-2 KD cells is interesting in light of previous reports showing on the one hand that malignant cells have less β-actin stress fibers than normal cells, and in contrast, display γ-actin stress fiber networks; and on the other, that enhanced γ-actin expression promotes neoplastic features such as enhanced cell migration (for review see [49]). In this respect, it is pertinent to mention that we previously reported that ZO-2 KD MDCK cells display a profusion of stress fibers and a basal accumulation of NMIIA, and that they move more than parental cells but with a reduced directional persistence [6]. 

In hZO-1, an actin-binding region (ABR) was previously reported [50]. Within it, an actin-binding site (ABS) was identified that displayed a weaker affinity for actin than other junctional proteins such as α-catenin, which resulted essential to maintaining the barrier function of the TJ [51]. ZO-2 associates with actin [4] through its carboxyl-terminal portion [5]. This segment of ZO-2 has an ABR resembling that of ZO-1, which contains a sequence of 12 amino acids with a 67% identity to the ABS of ZO-1 (Supplementary Figure S2). However, the affinity to actin of this putative ABS of ZO-2 has yet to be studied.

Here, we found that the tensile stress determined by FRET with the ZO-1 sensor increases in cells where ZO-2 is silenced, thus suggesting that ZO-2, through its heterodimerization with ZO-1, participates in maintaining mechanical stress at the TJ. ZO-1 is a mechano-sensing protein in either a stretched or folded conformation. The interaction between an amino-terminal motif in cingulin with the ZU5 domain of ZO-1 promotes the extended conformation, stabilization, and accumulation of ZO-1 at TJs [52]. The stretched conformation of ZO-1 promotes the junctional localization of occludin and the transcription factor ZONAB. However, this also depends on the presence of ZO-2. Thus, upon blebbistatin treatment, ZO-1 folding occurs only if ZO-2 is depleted, thus suggesting that heterodimerization of ZO-1 with ZO-2 stabilizes the stretched conformation of ZO-1 [53].

Tensile stress at the cell border is critical to repair TJ leaks. Thus, local breaches in barrier function along the cell border are restored by Rho flares that trigger actomyosin-mediated junction contraction and TJ protein recruitment to the damaged site [54]. The increase in TJ tension triggered by the absence of ZO-2 aligns with the augmented Rho/ROCK activation previously observed in ZO-2 KD MDCK cells [6]. The increased expression of p114RhoGEF has been observed in the border of cells lacking ZO-2 and might reflect the attempts of these monolayers that lack a TJ protein to repair barrier leaks. In this regard, it should also be mentioned that an increased paracellular flux of large molecules has been observed in MDCK ZO-1 and ZO-2 double KD cells [9,10], ZO-1 KD cells [13], and ZO-2 KD cells [55]. 

The reduced expression of JAM-A at the cell borders observed in ZO-2 KD cells agrees with a previous report showing that JAM-A associates directly with the ZO-2 PDZ-2 domain [7]. These observations prompted us to confirm, via in silico analysis, the direct interaction between ZO-2 PDZ-2 and the carboxyl-terminal segment of JAM-A. We computed hydropathy values of the JAM-A-tail and PDZ-2 domain complex as well as the PDZ-2 domain itself to determine the hydrophobic binding region, which matches the conformation in a similar crystal structure of the JAM-A tail in complex with ZO-1 PDZ-3 domain. The specific contacts contributing to this conformation’s stability were inspected using interprotein contact analysis with a 6.5 Å cutoff. These last three residues of the JAM-A are predominantly hydrophobic and bind the hydrophobic pocket of the ZO-2 PDZ-2 domain with a binding energy of −59.4 ± 1.75 kJ/mol. The stability and conformation of the JAM-A tail and ZO-2 PDZ-2 complex are consistent with the crystal structure of the JAM-A tail in complex with the ZO-1 PDZ-3 domain [56].

We have shown that the presence of ZO-2 is important for tethering JAM-A to the cell borders. The latter is critical for maintaining the TJ barrier to macromolecules, since MDCK cells with a quintuple KO of claudins have no junctional strands and lose the paracellular barrier to ions but preserve the barrier to larger molecules unless JAM-A is also deleted [33]. These observations, together with our findings, indicate that the presence of ZO-1 and ZO-2 is not only crucial for the polymerization of claudins and the establishment of a paracellular barrier for ions, as was previously shown [57], but also for the recruitment of JAM-A and the development of a paracellular macromolecular barrier.

Here, we observed that the lack of ZO-2 provokes vinculin accumulation at tTJs. This observation confirms a state of increased junctional tension in ZO-2 KD cells, as vinculin staining at tTJs is known to vary following the conformation state of α-catenin, which is regulated in turn by the contractile activity of NMII. Vinculin accumulation at tTJs is crucial, as it reinforces the connection of tricellulin to actomyosin via α-catenin, enabling the linkage of the converging bTJ strands with the central sealing element of tTJs [36].

Tension at the lateral membrane, modulated by the interaction of the E-cadherin/β-catenin/α-catenin complex with actomyosin, is not altered by the absence of ZO-2, indicating that ZO-2 regulates tension at the TJ and not the AJ level. These results tally with those obtained upon JAM-A depletion, which triggered an increase in apical tension while exerting no effect on AJ tension [15]. In contrast, the depletion of ZO-1 reduces tension on vascular endothelial (VE) cadherin in primary dermal microvascular endothelial cells [58]. This different outcome might be explained by considering that ZO-1 and ZO-2 share some functions but are not redundant, and also by the flat shape of endothelial cells, which provokes intermingling at the lateral membrane of TJs and AJs, making it hard to spatially distinguish TJs from AJs [59].

We also observed hole formation in ZO-2 KD cells plated on 20 kPa hydrogels. This result indicates high tension, as hole formation is not present in parental monolayers and has only been reported in MDCK cells cultured on soft 2.3–8.6 kPa gels [37]. Interestingly, those monolayers displayed an increased concentration of vinculin at intercellular junctions [37], which tallies with our observation of vinculin in ZO-2 KD cells. 

The stiffness of the ECM is a parameter that significantly impacts cell behavior (for review, see [38]). Accordingly, stiffening of the ECM within the intima of vessels during aging is a hallmark of atherosclerosis [60], and excessive extracellular matrix deposition in fibrotic diseases such as pulmonary fibrosis [61] and liver cirrhosis [62] leads to the stiffening of the epithelial tissue and organ dysfunction. In addition, the loss of stiffness sensitivity might allow the survival of metastatic cells as they invade tissues of different rigidities [63]. In this study, we investigated if the lack of ZO-2 altered the response of epithelial MDCK cells to the stiffness of the ECM with regard to cell elongation and the formation of cell aggregates.

We observed that cells without ZO-2 were still sensitive to matrix stiffness and coating, as no cellular elongation was detected in cells plated on 1 kPa hydrogels covered with fibronectin or collagen IV, independently of the presence of ZO-2. A similar lack of elongation was previously found in primary hepatocytes plated on 1 kPa hydrogels [64]. In addition, we observed that both parental and ZO-2 KD cells plated on 20 kPa hydrogels elongated more on collagen than fibronectin-coated surfaces. However, in comparison to parental cells, elongation was delayed in ZO-2 KD cells.

The retarded elongation of ZO-2 KD cells in 20 kPa hydrogels could be related to both the diminished expression of integrin β1 previously found in ZO-2 KD cells [6] and to the overactivity of RhoA/ROCK present in these cells, which might diminish their capacity to respond to further activation by stiff matrixes in resemblance to their lack of response to further RhoA activation triggered by lysophosphatidic acid (LPA) [6].

Both parental and ZO-2 KD cells aggregated less in 1 kPa than in 20 kPa hydrogels, in accordance with what was previously found in hepatocytes [64]. In addition, the cells formed more aggregates in the 20 kPa hydrogels covered with fibronectin than those covered with collagen. However, the formation of cell aggregates was delayed in ZO-2 KD cells. This result thus indicates that ZO-2 facilitates cell–cell adhesion, which is expected for a TJ protein, and agrees with a previous result showing that the lack of ZO-2 generates a widened intercellular space [55] and favors cell disaggregation after trypsin treatment [6]. 

Finally, we tested if the lack of ZO-2 affected the sensitivity of MDCK cells to the nanoscale topography of the extracellular matrix by plating parental and ZO-2 KD cells on arrays of nanoscale aligned fibers similar in size and organization to ECM fibers. We observed a more abundant presence of claudin-4 in cytoplasmic vesicles in ZO-2 KD cells in comparison to parental cells, indicating that the absence of ZO-2 in cells cultured on NRA provokes instability at the TJ, which favors protein internalization. We also found that the lack of ZO-2 is sufficient to induce YAP nuclear concentration, independently of cell density or substrate topography. This result aligns to our previous observations showing that in the absence of ZO-2, YAP concentrates at the nucleus in both confluent and sparse cultures [43]. ZO-2 associates to the Hippo pathway kinase LATS, promoting its activation and cell border recruitment [65]. LATS phosphorylates YAP, and this phosphorylation allows YAP sequestration in the cytoplasm by 14.3.3 protein, in consequence blocking YAP nuclear importation [43]. Therefore, the absence of ZO-2 facilitates YAP nuclear concentration.

In addition, we observed that the absence of ZO-2 favors Snail nuclear concentration, suggesting an accentuated sensitivity of the cells to substrate nanoscale topography for the development of a partial-EMT phenotype. 

## 4. Materials and Methods

### 4.1. Cell Culture

Parental (control) and ZO-2 KD MDCK II cells were generously provided by Alan Fanning (University of North Carolina, Chapel Hill, NC, USA) and cultured as previously described [13]. These cells stably express a mixture of three different shRNAs against ZO-2 in the pSuper vector. Parental cells only express the empty vector instead. The stable clonal ZO-2 KD MDCK cell line employed (IC5) was obtained based on zeocin resistance. Supplementary Figure S3 illustrates, via immunofluorescence and Western blot, that ZO-2 KD cells do not express detectable levels of ZO-2.

Parental and ZO-2 KD MDCK monolayers were plated at confluent (5 × 10^5^ cells/cm^2^) or sparse (4 × 10^4^ cells/cm^2^) density on glass coverslips, hydrogels, or nanostructured ridge arrays (Circular NanoSurface coverglass Cat. No. ANFS-CS12, Curi Bio, Seattle, WA, USA). The latter are coverglasses covered with a nanopattern of submicron grooves and ridges formed by a very thin layer of polymer.

### 4.2. Measurement of Apical Membrane Rigidity with Atomic Force Microscopy

Atomic force microscopy was employed to measure the rigidity of the apical membrane of parental and ZO-2 KD MDCK cells. Young’s module was determined with an atomic force microscope (BioScope Catalyst AFM, Bruker, Billerica, MA, USA) equipped with a fluid chamber and a thermally controlled AFM plate, where the epithelial monolayer plated at confluent density (2.3 × 10^6^ cells/cm^2^) on a glass slide was kept at 37 °C, bathed with PBS with or without 1.8 mM CaCl^2^ (Figure 1A). For the low-calcium (LC) condition, monolayers were washed twice with calcium-free PBS (LC-PBS) and incubated for 20 min in LC-PBS with 2.4 mM EDTA (Cat. 3002E, Research Organics, Cleveland, OH, USA) before the indentation procedure. Then, the monolayers were incubated in LC-PBS and kept in this medium throughout the indentation procedure. Another group of monolayers was kept in the incubator for 15 h with CDMEM containing 100 µM blebbistatin (Cat. 674289-55-5, TOCRIS Bioscience, Bristol, UK) or for 4 h with CDMEM containing 10 µM docetaxel (Cat. 01885, Merck Millipore, Darmstadt, Germany). Then, those monolayers were washed twice with PBS, transferred to the fluid chamber sealed by an O-ring, and maintained during the indentations with PBS with 100 µM blebbistatin or 10 µM docetaxel.

Indentation was conducted with the cantilever DNP-10A (Bruker, Billerica, MA, USA) with a 20 nm tip radius (Bruker, Billerica, MA, USA). The cantilever’s spring constant was calculated with the thermal tune method, which employs the value of deflection sensitivity obtained with a force-distance curve acquired on a stiff glass slide. Apical rigidity was measured with the point-and-shoot (TM) mode in 5 × 5 µm areas with 5 nN force and 6.7 µm/s velocity. Cell surface areas were randomly selected, and, in each area, 100 measurements were made using 10 × 10 indentation square matrices.

The Young module in kPa was obtained with data processed by NanoScope Analysis software (Bruker 1.40). A baseline correction was performed for each force curve, which was then fitted with the Sneddon model for conical cantilever tips with the following equation:

(1) $$F=\frac{2}{\pi }\;\frac{E}{\left(1-v^{2}\right)}\tan\left(\alpha \right)\delta^{2}$$

where the force (F) in nN was obtained from force curves (Figure 1B), Young’s modulus is E (kPa), v is Poisson’s ratio (typically 0.2–0.5; for cell experiments v = 0.5 was used), α is the half-angle of the indenter (18°), and δ is the indentation distance (nm). 

In the graph in Figure 1C, each dot plot represents one value of Young’s modulus obtained from one force-indentation curve with an R^2^ ≥ 0.98. The total number of fitted force-indentation curves used to generate the figure is indicated.

### 4.3. Modeling of ZO-2/JAM-A Complex

We adopted a stepwise approach to building the structure of PDZ-2 ZO-2 and JAM-A tail complex on the structural framework of a previous study, which showed the binding of the last ten residues (EFKQTSSFLV) of human JAM-A tail to the ZO-1 PDZ-3 and SH3 domains (PDB ID: 3TSZ) [56]. First, we built the ZO-2 PDZ-2 model using Alphafold based on the UniProt (:Q9UDY2) sequence [49,66]. Specifically, we utilized the sequence 306GVLLMKSRANEEYGLRLGSQIFVKEMTRTGLATKDGNLHEGDIILINGTVTENMSLTDARKLIEKSRGKLQLVVLRDSQ386 to represent the ZO-2 PDZ-2 structure from the full ZO-2 protein. The Alphafold confidence of the predicted PDZ-2 domain gave scores in the confident or very high region for most of the segment used. Only two residues were found in the “Low” category, namely S385 and Q386 with confidence scores of 67.5 and 60.0, respectively [66]. Second, we used the 3TSZ crystal structure as a template to align the predicted ZO-2 PDZ-2 structure on the ZO-1 PDZ3 domain in PyMOL [67]. Third, we deleted the ZO-1 PDZ-3 and other components in 3TSZ, leaving the EFKQTSSFLV residues of the JAM-A tail and the ZO-2 PDZ-2 domain for further characterization. Next, using the CHARMM-GUI web server [68,69,70], the JAM-A/ZO-2 PDZ-2 complex was placed in a cubic box of 10 nm length for energy minimization and equilibration using molecular dynamics (MD) simulations. The simulation box contained 32,129 water molecules, 90 Na^+^ and 93 Cl^−^ to neutralize the system’s charge and maintain a 0.15 M ionic solution. 

The proteins and ions were modeled using Charmm36 forcefield parameters, and water was modeled using TIP3P parameters [71]. This system was simulated in the GROMACSv2019.4 simulation package [72]. Energy minimization utilized the steepest-descent algorithm for 5000 steps, followed by isothermal-isochoric (NVT) and isothermal-isobaric (NPT) equilibration, followed by a production stage of 70 ns with a 2 fs timestep in NPT ensemble. Equilibration and production steps used the velocity-rescale thermostat [73] at 310.15 °K and a τ_T_ of 1.0 ps. For production, we used a Verlet cutoff of 1.2 nm, particle mesh Ewald algorithm [74,75] at 1.2 nm cutoff, velocity rescale thermostat of the same parameters, and Parrinello-Rahman barostat [76] with isotropic pressure coupling at 1 bar, compressibility at 4.5 × 10^−5^ bar^−1^, and a τ_p_ of 5.0 ps. Position restraints were placed on the entire system during minimization and equilibration. In the production run, the position restraints were placed on the PDZ domain to prevent loss in the secondary structure. No constraints were placed on the JAM-A tail.

### 4.4. PARCH Scale Calculations

Following equilibration, PARCH scale calculations [28] were performed on the complex. The protein complex was extracted and placed into a new simulation box, surrounded by a shell of water with a thickness of *d*_shell_ = 0.415 nm. The cubic box length was set at 16.25 nm, derived from the equation *l* = 2 × (*d*_max_ + *d*_ion_ + *d*_b_), where *d*_max_ = 3.125 nm is the maximum radius of the protein, *d*_ion_ = 3.0 nm is the distance of the hydrated counter ions from the protein’s surface, and *d*_b_ = 3.0 nm is the distance between the hydrated ions and the periodic cell boundary. The MD annealing process was used to increase the temperature from 300 to 800 °K with an annealing rate of 1 K/10 ps at constant volume. Water molecules contacting residues were calculated with a 0.315 nm cutoff. During the annealing process, the protein complex and counter ions were position-restrained with a force constant of 1000 kJ mol^−1^ nm^−2^ to ensure the protein’s conformation and distance of the counter ions remained constant. The PARCH calculations were performed in triplicate, and the average value of the three runs was taken for the final report. The process was repeated for the PDZ-2 domain without the JFAM-A tail.

### 4.5. Contact Analysis

The final structure conformation obtained from the 70 ns production run was used for contact analysis between PDZ-2 and JAM-A proteins, using a cutoff distance of 6.5 Å from the center of geometry (COG) of each residue in PDZ-2 to the COG of the residues in JAM-A tail. The contact analysis was conducted using in-house Python 3.7.12 scripts, the MDAnalysis package 2.1.0 [77,78], and qualitative inspection using PyMOL 2.3.3 [67].

### 4.6. Steered Molecular Dynamics

The binding energy of the JAM-A 10-mer tail with the ZO-2 PDZ-2 domain was determined using steered molecular dynamics with the GROMACS 2022.2 version [72,79]. Steered MD is a standard molecular dynamics technique for computing the binding energy between interacting molecules. This approach applies a constant force to specific groups of atoms while restraining the remaining system. This application of the force induces an unbinding event. In this study, the backbone and sidechains of the PDZ-2 domain were restrained with a 4 kJmol^−1^ nm^−1^ force constant. The 10-mer was then pulled perpendicular to its resting position to remove it from the PDZ domain and not cause any new interactions during the pull. This process used a constant harmonic force of 800 mol^−1^ nm^2^ and a pull rate of 1.25 × 10^−3^ nm ps^−1^. Uniformly spaced windows at 0.05 nm were selected for umbrella sampling simulations. The binding energy was computed using the GROMACS 2022.2 [72] weighted histogram analysis method [79,80] and the Bayesian bootstrap method. 

### 4.7. FRET Analysis and Quantification

#### 4.7.1. Constructs

The E-Cadherin tension sensor (E-cad TSMod) and corresponding control constructs employed here were previously described [31] and generously provided by Dr. Alexander R. Dunn (Stanford University). This tension sensor was generated with a variant of canine E-cadherin that contained a tension sensor module (TSMod) with TFP and YFP fluorophores separated by an elastic linker derived from spider silk. This module was inserted in the cytoplasmatic domain between the transmembrane domain and the β-catenin binding domain. E-cad TSModΔcyto is a control construct where the β-catenin binding domain was deleted to achieve maximum FRET. E-cad TSModΔYFP construct only contains TFP as a fluorophore, and the E-cad TSModΔTFP construct only contains the YFP fluorophore. 

ZO-1 tension sensor (ZO-1 TS) and the corresponding control construct employed here were previously described [15] and generously provided by Maria S. Balda (University College, London, UK). ZO-1 TS was constructed by inserting a FRET module with CFP and YFP fluorophores connected with an elastic linker derived from spider silk protein. This FRET module was located between the ZO-1 N-terminal motifs that interact with junctional partners of ZO-1 and the C-terminal domain (CTD), which includes the actin-binding region. ZO-1 TS-ΔCTD control construct contains the FRET module but lacks the CTD. Therefore, the ZO-1 TS-ΔCTD construct cannot sense tensile stress.

#### 4.7.2. Image Acquisition 

Parental and ZO-2 KD MDCK cells seeded at subconfluence on glass coverslips were transfected with E-Cadherin or ZO-1 tension sensors with Lipofectamine 2000™ (Cat. 11668-019, Life Technologies, Eugene, OR, USA). Some monolayers were treated for 15 h with 100 µM blebbistatin (Cat. B0560, Sigma Aldrich, Saint Louis, MO, USA), 6 h after their transfection with the tension sensor constructs. Upon reaching confluency, the monolayers were fixed with paraformaldehyde 4% (*v*/*v*) for 10 min at room temperature and mounted with Vectashield (Cat. No. H-1000, Vector Laboratories Inc., Burlingame, CA, USA). Images were acquired in a Leica SP8 confocal microscope as follows: For the donor channel (emission of the donor upon excitation of the donor), TFP or CFP were exited with the argon laser line at 462 nm, and the emission window was collected between 470–500 nm. For the acceptor channel (emission of the acceptor upon excitation of the acceptor), YFP was exited with the argon laser line at 514 nm, and the emission window was collected between 530–600 nm. Finally, for the FRET channel (emission of the acceptor upon excitation of the donor), TFP or CFP were exited with the argon laser line at 462 nm and the emission window collected between 530–600 nm. 

#### 4.7.3. FRET Quantification

In every image, all the channels were equally background-subtracted. The spectral bleed-through coefficient was determined using the images of cells expressing E-cad TSModΔYFP or E-cad TSModΔTFP constructs taken in donor, acceptor, and FRET channels by calculating the mean fluorescence intensity of junctional segments manually selected with the polygon selections tool of ImageJ version 1.54f. from two adjacent transfected cells. The spectral bleed-through donor (SBTdonor) and acceptor (SBTacceptor) were calculated with the following equations [63]:

(2) $$SBTdonor=\frac{IFRET}{Idonor}$$

(3) $$SBTacceptor=\frac{IFRET}{Iacceptor}$$

where IFRET is the mean fluorescence intensity from the FRET channel; Idonor is the mean fluorescence intensity from the donor channel; and Iacceptor is the mean fluorescence intensity from the acceptor channel.

The images of the E-cadherin and ZO-1 tension sensors were then used to obtain the mean fluorescence intensity. To correct for spectral bleed-through in experimental data, the following equation was used [63]:

(4) $$cFRET=IFRET-SBTdonor\left(Idonor\right)Idonor-SBTacceptor\left(Iacceptor\right)Iacceptor$$

where cFRET is the corrected FRET; IFRET is the mean fluorescence intensity from the FRET channel; SBTdonor is the donor spectral bleed-through; SBTacceptor is the acceptor spectral bleed-through; Idonor is the mean fluorescence intensity from the donor channel; and Iacceptor is the mean fluorescence intensity from the acceptor channel.

Finally, the cFRET values were normalized for variations in intensity and reported as a FRET index with the following equation [63]:

(5) $$FRETindex=\frac{cFRET}{Iacceptor}$$

where cFRET is the corrected FRET and Iacceptor is the mean fluorescence intensity from the acceptor channel from the E-cadherin and ZO-1 tension sensors.

The representative FRET images were obtained using the FRET and Colocalization Analyzer plug-in of ImageJ version 1.54f [81].

### 4.8. Western Blots

Western blots were conducted according to standard procedures as previously reported [82], using the following rabbit polyclonal antibodies against p114RhoGEF (Cat. No. 102223, dilution 1:1000, GeneTex, Irvine, CA, USA), anti JAM-A (Cat. No. 361700, dilution 1:1000, Life Technologies, Carlsbad, CA, USA), anti afadin (Cat. No. A0224, dilution 1:2000, Sigma Aldrich, St. Louis, MO, USA), and anti ZO-2 (Cat. No. 711400, dilution 1:500, Invitrogen, Carlsbad, CA, USA); or mouse monoclonals against vinculin (Cat. No. 13901, dilution 1:1000, Cell Signaling, Danvers, MA, USA), β-actin (Cat. MAS-15739, dilution 1:5000, Invitrogen, Waltham, MA, USA), and γ-actin (Cat. Sc-65638, dilution 1:5000, Santa Cruz Biotechnology, Dallas, TX, USA). As secondary antibodies, we employed peroxidase-conjugated goat antibodies against rabbit IgG (Cat. 62-6120, dilution 1:20,000, Invitrogen, Camarillo, CA, USA) or mouse IgG (Cat. 62-6520, dilution 1:10,000, Invitrogen, Camarillo, CA, USA) and a chemiluminescence detection system Immobilon™ western (Merck Millipore, Cat. WBKLS 0500, Darmstadt, Germany).

To obtain soluble and insoluble fractions, we employed a previously reported protocol [83]. In brief, monolayers of parental and ZO-2 KD cells were scraped into a buffer with 120 mM NaCl, 25 mM HEPES pH 7.5, 2 mM EDTA, 25 mM NaF, 1 mM NaVO_4_, 1% (*w*/*v*) Triton X-100 and 5% (*v*/*v*) of the protease inhibitor cocktail Complete (Cat. 11697498001, Roche, Mannheim, Germany). After 30 min on ice, the lysate was centrifuged at 13,000 rpm for 30 min at 4 °C. The supernatant containing the detergent-soluble fraction was retained. At the same time, the detergent-insoluble pellet was solubilized by sonication for 3 s on ice in a buffer with 25 mM HEPES pH 7.5, 2 mM EDTA, 25 mM NaF, 1 mM NaVO_4_, 5% (*v*/*v*) of protease inhibitor cocktail and 1% (*w*/*v*) sodium dodecyl sulfate (SDS). The protein content of both detergent soluble and insoluble fractions was determined, and fractions were stored until used at −80 °C.

### 4.9. Immunofluorescence

To achieve optimal immunofluorescence images of different proteins, we employed various fixation, permeabilization, blockade, and antibody solutions and protocols according to the antibodies employed (Table 2) as previously described [22,84]. Then, all monolayers were washed thrice with PBS and mounted with Vectashield/DAPI (Cat. No. H-1200, Vector Laboratories Inc., Burlingame, CA, USA). For the immunofluorescent detection of F-actin, we additionally employed rhodaminated phalloidin (Cat. No. R415, dilution 1:500, Invitrogen, Eugene, OR, USA), which was added to monolayers for 2 h at the same time as the monolayers were incubated with the secondary antibody against vinculin.

As secondary antibodies, we employed donkey antibodies against rabbit IgG coupled to Alexa Fluor 488 (Cat. A-21206, dilution 1:1000, Life Technologies, Eugene, OR, USA) or Alexa Fluor 594 (Cat. A-21207, dilution 1:1000, Life Technologies, Eugene, OR, USA); donkey antibodies against mouse IgG coupled to Alexa Fluor 488 (Cat. A-21202, dilution 1:1000, Invitrogene, Eugene, OR, USA) or Alexa Fluor 594 (Cat. A-21203, dilution 1:1000, Life Technologies, Eugene, OR, USA); and donkey antibodies against rat IgG coupled to Alexa Fluor 488 (Cat. A-21208, dilution 1:1000, Life Technologies, Eugene, OR, USA).

### 4.10. Proximity Ligation Assay 

Proximity ligation assays (PLA) were conducted following the manufacturer’s instructions (PLA Duolink, Cat. DUO92008, Sigma Aldrich, Uppsala, Sweden) in parental and ZO-2 KD MDCK cells with mouse antibodies against γ-actin (Cat. sc-65638, dilution 1:200, Santa Cruz Biotechnology, Dallas, TX, USA) and rabbit antibodies against ZO-1 (Cat. 61-7300, dilution 1:100, Invitrogen, Camarillo, CA, USA). For background detection, parental cells were incubated only with the antibody against ZO-1.

### 4.11. Polyacrylamide Hydrogels with Soft and Stiff Elastic Moduli

Polyacrylamide hydrogels were generated as previously described [64,85] with minor modifications. Briefly, for the preparation of hydrogels, two mixes of degasified acrylamide/bis-acrylamide were made: one for soft (1 kPa) hydrogels with 3% acrylamide (Cat No. A4058, Sigma Aldrich St. Louis, MO, USA) and 0.1% N, N’-methylene bis-acrylamide (Cat No. M1533, Sigma Aldrich, St. Louis, MO, USA), and another for stiff (20 kPa) hydrogels with 8% acrylamide and 0.264% N, N’-methylene bis-acrylamide. Next, 10% ammonium persulfate and 1% TEMED were added to each mixture. Then, 90 μL of these mixtures were immediately placed on top of a glass slide previously treated with dichlorodimethylsilane (Cat No. 440248, Sigma Aldrich St. Louis, MO, USA) to generate a non-adherent surface. The mixtures were then covered with a 20 mm round glass coverslip previously treated with 50 μL of (3-Aminopropyl) triethoxysilane (APTES) (Cat No. 440140, Sigma Aldrich, St. Louis, MO, USA)/glutaraldehyde to generate an adherent surface. After polymerization for a minimum of 25 min at RT, the hydrogels were treated with 0.5 mM sulfosuccinimidyl-6-(4′-azido-2′-nitrophenylamino)-hexanoate (sulfo-SANPAH) (Cat No. 803332, Sigma Aldrich, St. Louis, MO, USA) in 50 mM HEPES pH 8.2 and 25% DMSO, whose crosslinker activity was then activated by UV radiation at 360 nm for 10 min. The hydrogels were then washed thrice with 50 mM HEPES pH 8.2. Human fibronectin (Cat. 356008, Corning, Bedford, MA, USA) or mouse collagen IV (Cat. 354233, Corning, Bedford, MA, USA) at a concentration of 20 μg/mL in HEPES 50 mM pH 8.2 were placed on top of the sulfo-SANPAH crosslinked polyacrylamide hydrogels and left ON at 4 °C. Then, the hydrogels were washed thrice with HEPES 50 mM pH 8.2. In representative samples of these hydrogels, their rigidity was measured with a microindenter with a glass sphere of 50 μm in diameter mounted on a force sensor, as previously described [86]. The rest of the gels were sterilized by UV irradiation for 10 min and then employed as substrate for plating parental and ZO-2 KD MDCK cells. 

### 4.12. Quantitation of Cell Area and Elongation 

Cell area and elongation were quantitated with ImageJ using the Analyze option. Then, the following steps were followed: First, measurements/area or shape descriptors were set. Next, with the polygon tool, the cells were manually selected, and after clicking Measure, the numerical values of area and aspect ratio were obtained. Cell elongation was calculated as aspect ratio = [Major Axis]/[Minor Axis].

The percentage of round cells was calculated after manually counting the number of round cells with ImageJ version 1.54f using the Multi-point tool.

### 4.13. Drugs

Docetaxel (Cat. 01885, Merck Millipore, Darmstadt, Germany) was prepared as a 10 mM stock in DMSO and used at a concentration of 10 μM. Blebbistatin (Cat. B0560, Sigma Aldrich, Saint Louis, MO, USA), was prepared as a 10 mM stock in DMSO and used at a concentration of 100 μM. 

## 5. Conclusions

ZO-2 acts as a modulator of mechanical tension in epithelial cells. The absence of ZO-2 in MDCK cells leads to a reduced stability of microtubules and a reduction of γ-actin concentration at the TJ belt, which might explain the decreased apical membrane tension observed. In silico analysis confirms a stable interaction of ZO-2 PDZ-2 domain with the JAM-A tail. Accordingly, the lack of ZO-2 in MDCK cells inhibits JAM-A tethering to the cell borders, leading to the TJ accumulation of p114RhoGEF and afadin and an increased tension at bicellular and tricellular TJs but not at AJs. This increase in TJ tension in cells lacking ZO-2 favors the formation of holes in cells plated in 20 kPa hydrogels. ZO-2 absence also retards cell elongation and cell aggregates formation in monolayers plated in hydrogels and exacerbates the partial transformation triggered by substrate topography.

## Figures and Tables

**Table 1:** Parch values and residue contacts of JAM-A C-terminal tail with ZO-2 PDZ-2 domain. The JAM-A residues are represented backwards from the C-terminal with the associated sequence of EFKQTSSFLV, where C-1 V denotes the first residue from the C-terminal. The cutoff for a registered contact between center of geometries of each residue is 6.5 Å.

JAM-A			ZO-2 PDZ-2 ^a^
Position	Residue	Parch Values	Contacts
C-1	V	0.9	L321 L323 I370 R367
C-2	L	0.4	R322
C-3	F	0.1	G324 L363 R367
C-4	S	0.2	R322 K330 E331
C-5	S	0.0	-
C-6	T	0.2	-
C-7	Q	0.1	G347 S325 K330
C-8	K	1.3	-
C-9	F	0.3	Q326 F328
C-10	E	2.2	G347

^a^ ZO-2 PDZ-2 sequence: 306-IGVLLMKSRANEEYGLRLGSQIFVKEMTRTGLATKDGNLHEGDIILKINGTVTENMSLTDARKLIEKSRGKLQLVVLRDSQ-386.

**Table 2:** Protocols for immunofluorescence detection of different proteins.

Antibody	Fixation	Permeabilization	Blockade	1st Antibody ON Incubation Solution	2° Antibody 2 h Incubation Solution
Rabbit α NMM IIB (Cat. 909901, dilution 1:200, BioLegend, San Diego, CA, USA).	PFA 1% (*v*/*v*), 12 min, RT ↓ methanol 100% (*v*/*v*), −20 °C, 5 min	Triton X-100 0.2% (*v*/*v*), 5 min, RT	BSA 2% (*w*/*v*), 30 min, RT	BSA 2% (*w*/*v*), 4 °C	BSA 1% (*w*/*v*), RT
Mouse α β-actin (Cat. MA5-15739, dilution 1:100, Invitrogen, Waltham, MA, USA). Mouse α γ-actin (Cat.sc-65638, dilution 1:200, Santa Cruz Biotechnology, Dallas, TX, USA). Rat α ZO-1 (Cat. R26.4C, dilution 1:10, DSHB, University of Iowa, IA, USA). Mouse α vinculin * (Cat. V4505, dilution 1:100, Sigma Aldrich, St Louis, MO, USA).	PFA 4% (*v*/*v*), 10 min, RT ↓ methanol 100% (*v*/*v*), −20 °C, 5 * to 15 min		ASE blocking solution, 10 to 30 * min, RT	BSA 1% (*w*/*v*), 4 °C	
Rabbit α afadin (Cat. A0224, dilution 1:400 Sigma Aldrich, St. Louis, MO, USA). Rabbit α YAP (dilution 1:1000, generously provided by Marius Sudol, Mechanobiology Institute, National University of Singapore). Rabbit α snail * (Cat. GTX125918, dilution 1:100, GeneTex, Irvine, CA, USA). Rabbit α ZO-2 (Cat. 71-1400, dilution 1:100, Invitrogen, Waltham, MA, USA).	PFA 4% (*v*/*v*), 10 min, RT	Triton X-100 0.5% (*v*/*v*), 10 to 15 * min, RT	BSA 1% (*w*/*v*), RT		
Mouse α claudin-4 * (Cat. 329400, dilution 1:200 Invitrogene, Camarillo, CA, USA). Rabbit α p114RhoGEF (Cat. 102223, dilution 1:50, GeneTex, Irvine, CA, USA). Rabbit α JAM-A * (Cat. 361700, dilution 1:100, Life Technologies, Carlsbad, CA, USA).	methanol 100% (*v*/*v*), −20 °C, 10 * to 20 min	Triton X-100 0.2% (*v*/*v*), 10 min, RT	ASE blocking solution, 10 to 20 min, RT		

RT, room temperature; ON, overnight; ASE, (Antibody Signal Enhancer) blocking solution [50 mM glycine, 0.1% Triton X-100 (*v*/*v*), 0.1% BSA (*v*/*v*), 0.05% Tween 20 (*v*/*v*), and 2% horse serum (*v*/*v*]; BSA, IgG-free bovine serum albumin (Cat. No. 1331A, Research Organics, Cleveland, OH, USA). Asterisks (*) in antibodies relate them with the precise fixation, permeabilization or blockade times employed.

## Data Availability

Data are contained within the article and supplementary materials.

## Supplementary Materials

The following supporting information can be downloaded at: https://www.mdpi.com/article/10.3390/ijms25052453/s1.
