# Supplementary material for: The Role of ZO-2 in Modulating JAM-A and γ-Actin Junctional Recruitment, Apical Membrane and Tight Junction Tension, and Cell Response to Substrate Stiffness and Topography
Source: Int J Mol Sci. 2024 Feb 20;25(5):2453. doi: 10.3390/ijms25052453 (PMC10930961; doi:10.3390/ijms25052453)
Supplement: Supplementary file 1 [file ijms-25-02453-s001.zip › ijms-2769431-supplementary.pdf]

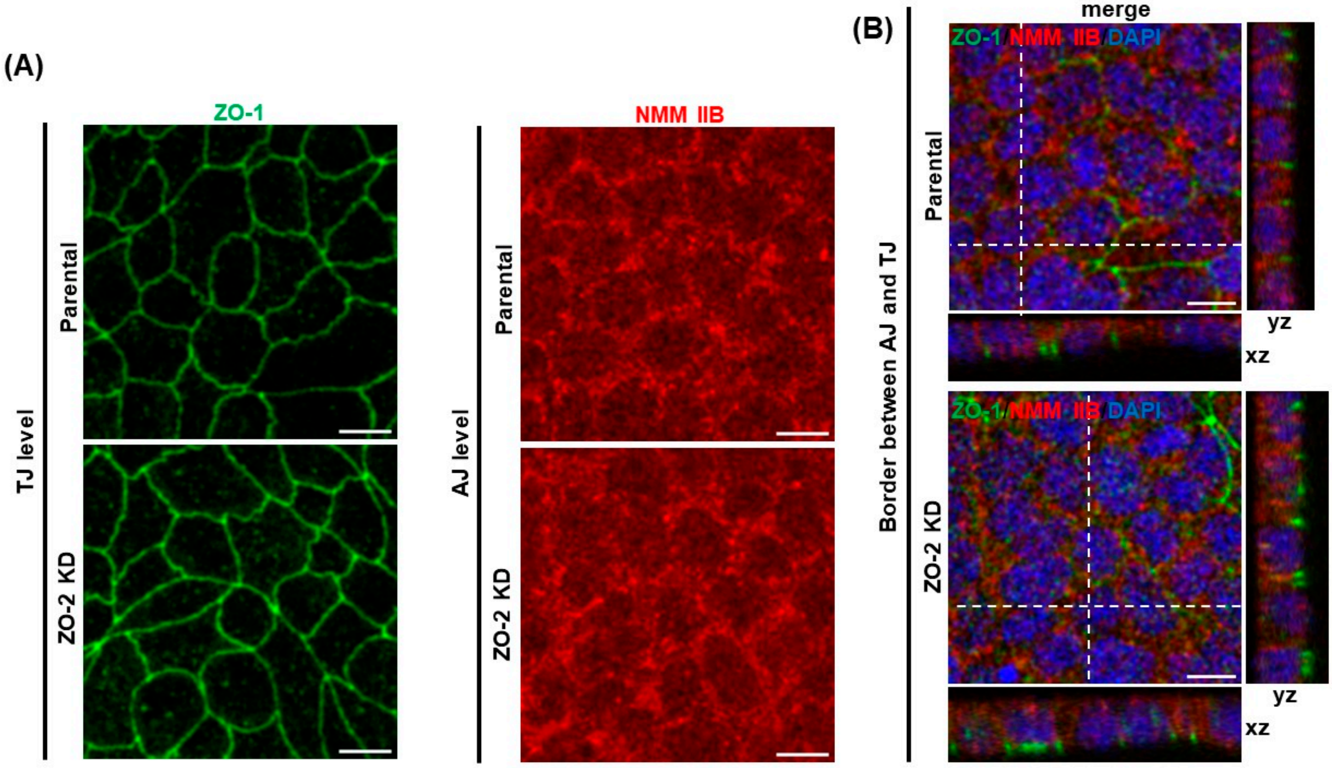

Supplementary Figure S1. The lack of ZO-2 does not affect NMM IIB concentration at the AJ region of epithelial MDCK cells. Monolayers of parental and ZO-2 KD MDCK cells were processed for immunofluorescence with antibodies against ZO-1 and NMM IIB. (**A**) Representative images of two independent experiments. Pile-ups of confocal sections taken at the TJ or the AJ level and done with image J. (**B**) Merged images done with ImageJ of confocal sections taken at the limit between the TJ and the AJ. Confocal sections in xz and yz planes are made along the white dotted lines. Bars, 10 µm.

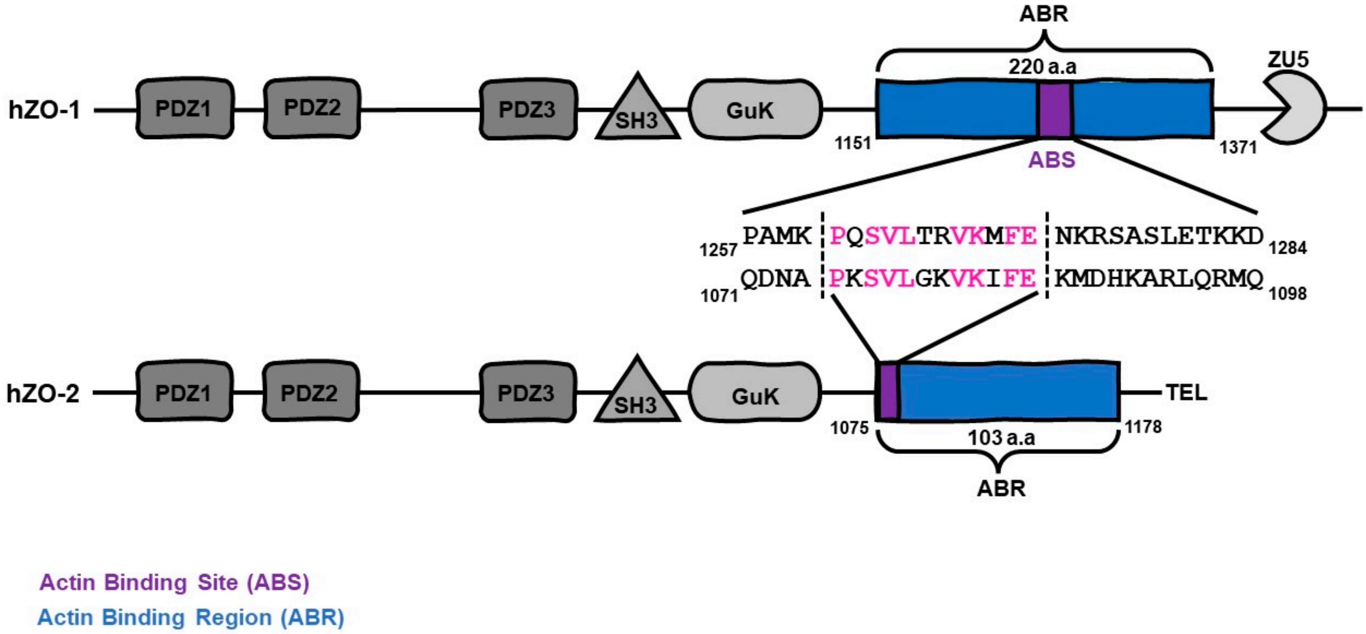

Supplementary Figure S2. hZO-2 contains a putative actin-binding region similar to the one in hZO-1. Scheme illustrating a BLAST search (NCBI) that compares the actin-binding region (ABR) and actin-binding sites (ABS) present in hZO-1 (Uniprot: Q07157) with the putative ones in hZO-2 (Uniprot: Q9UDY2).

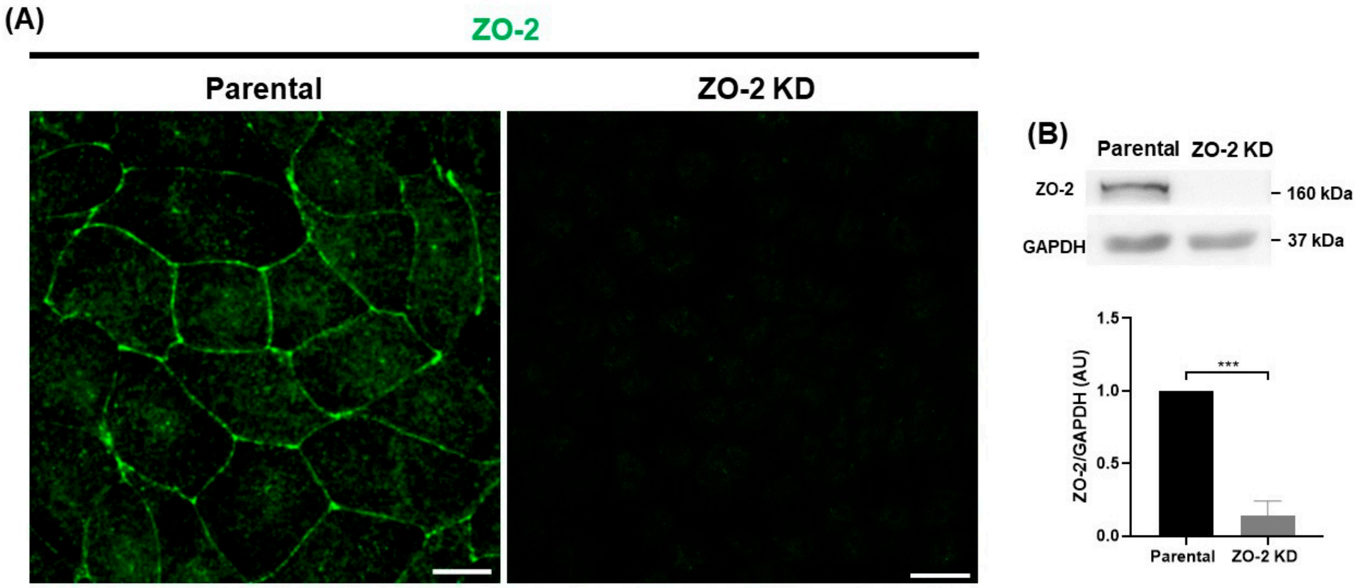

Supplementary Figure S3. ZO-2 cannot be detected in ZO-2 KD cells by immunofluorescence or Western blot. (**A**) Representative image of ZO-2 immunofluorescence in parental and ZO-2 KD cells. (**B**) Western blot detection of ZO-2 in parental and ZO-2 KD cells. GAPDH was employed as loading control. Statistical analysis was done with Student's t-test. Results shown as media ± standard deviation, *** $p < 0.001$.
